# MCL-1 as a molecular switch between myofibroblastic and pro-angiogenic features of breast cancer-associated fibroblasts

**DOI:** 10.1038/s41419-025-07920-6

**Published:** 2025-08-09

**Authors:** Chloé C. Lefebvre, Philippine Giowachini, Jennifer Derrien, Maxime Naour, Isabelle Corre, Laura Thirouard, Elise Douillard, David Chiron, François Guillonneau, Lucas Treps, Mario Campone, Philippe P. Juin, Frédérique Souazé

**Affiliations:** 1 Université de Nantes, INSERM, CNRS, CRCI2NA, Nantes, France; 2 Equipe labélisée LIGUE Contre le Cancer, Paris, France; 3 SIRIC ILIAD, Nantes, Angers, France; 4 Nantes Université, INRAE UMR1280, PhAN, IMAD, Nantes, France; 5 PROT’ICO - Plateforme Oncoprotéomique, Institut de Cancérologie de l’Ouest (ICO), Angers, France; 6 ICO René Gauducheau, Saint Herblain, France

**Keywords:** Apoptosis, Cancer microenvironment, Mitochondria

## Abstract

Breast cancer-associated fibroblasts (bCAFs) comprise inflammatory CAFs (iCAFs), characterized by the secretion of pro-inflammatory cytokines, and myofibroblastic CAFs (myCAFs), distinguished by their high production of extracellular matrix and their immunosuppressive properties. We previously showed that targeting the anti-apoptotic protein MCL-1 in primary culture of bCAF derived directly from human samples reduces their myofibroblastic characteristics. We herein show by single-cell RNA-sequencing analysis of bCAFs that MCL-1 knock down induces a phenotypic shift from wound-myCAF to IL-iCAF, characterized by the upregulation of genes associated with inflammation as well as angiogenesis-related genes. In vitro, genetic and pharmacologic MCL-1 inhibition increases VEGF secretion by bCAFs, enhancing endothelial cell tubulogenesis. In a chicken chorioallantoic membrane (CAM) model *in ovo*, co-engraftment of breast cancer cells and bCAFs with reduced MCL-1 expression leads to heightened peritumoral vascular density, driven by VEGF. Mechanistically, the pro-angiogenic phenotype revealed by MCL-1 inhibition is dependent on BAX-BAK activity. It results in NF-κB activation, inhibition of which by a IKKβ inhibitor suppresses the transcription of VEGF and pro-inflammatory factors triggered by MCL-1 inhibition in bCAFs. Chemotherapy downregulates MCL-1 in bCAFs via an increase of NOXA, the endogenous MCL-1 inhibitor, promoting a pro-angiogenic and inflammatory phenotype through the NOXA/MCL-1/NF-kB axis. Overall, these findings uncover a novel regulatory function of MCL-1 in determining bCAF subpopulation differentiation and highlight its role in modulating their pro-angiogenic properties, in response to treatment in particular.

## Introduction

Breast cancer-associated fibroblasts (bCAFs) are a predominant cellular component of the tumor stroma in breast cancers. Studies have identified a significant association between the abundance of bCAFs within tumors and unfavorable patient outcomes, underscoring their potential as therapeutic target [1–3]. Single-cell transcriptomic analysis have identified two primary populations of bCAFs, namely inflammatory CAFs (iCAFs) and myofibroblastic CAFs (myCAFs), each comprising three distinct clusters. iCAFs are known for their extensive production of pro-inflammatory soluble factors like IL-6, CXCL12, CXCL1, and others that can attract immune cells on the tumor site and include subsets defined by specific functional pathways characterized by detoxification processes (Detox-iCAF), associated with interleukin signaling (IL-iCAF), and linked to Interferon γ-mediated responses (IFNγ-iCAF). myCAFs are associated with a high contractile cytoskeleton linked to their pro-invasive features [4, 5] and an important role in the production and organization of the extracellular matrix (ECM) [6, 7]. In fact, the three myCAF clusters are characterized by a high expression of genes coding ECM proteins (ECM-myCAF), TGFβ signaling pathway (TGFβ-myCAF), or wound healing (Wound-myCAF) [7]. Interestingly, Detox-iCAFs serve as precursors to both iCAFs and myCAFs. The Wound-myCAF state represents a transient and less differentiated intermediate between Detox-iCAF and ECM-myCAF, acting as a bridge to more specialized subtypes, including ECM-myCAF and IFNαβ-myCAF, as revealed by trajectory analyses [8]. A spatial transcriptomic study showed that iCAFs are mainly found in the peritumoral stroma, while myCAFs are located in close contact with cancer cells [8].

Numerous studies have previously shown that CAFs promote tumor angiogenesis [9–11], cancer cell proliferation, survival, and therapy resistance [6, 7, 12, 13] in addition to metastases development [14–16]. Treatment resistance can be mediated, in part, by the altered balance between pro- and anti-apoptotic expression of the members of the BCL-2 family (with BCL-2, BCL-xL and MCL-1 being the main anti-apoptotic proteins) [17]. We previously demonstrated that bCAFs confer resistance to apoptosis in breast cancer cells through paracrine signaling by inducing overexpression of the anti-apoptotic protein MCL-1 [18]. Targeting MCL-1 in breast cancers appeared to be a promising strategy, especially as we identified it as the most commonly expressed anti-apoptotic protein in bCAFs as well [18]. We showed that targeting MCL-1 in bCAFs leads to non-lethal mitochondrial fragmentation accompanied by a loss of their myofibroblastic characteristics and pro-invasive abilities suggesting a role for MCL-1 in bCAFs plasticity [19].

MCL-1 is an essential regulator of mitochondrial homeostasis, mainly through its role in maintaining mitochondrial membrane integrity. Disturbances in this integrity influence various cellular processes, including metabolism, proliferation, and apoptosis [20]. Recent studies have also established a link between mitochondrial integrity and the regulation of inflammatory pathways [21–23]. Which of these MCL-1 functions contribute to the myofibroblastic features of bCAFs, and the underlying mechanisms, have remained elusive so far. In this study, we used genetic engineering of primary CAFs, single RNA sequencing, and a whole set of in vitro and in ovo explorations. We show that MCL-1 maintains the myofibroblastic features of CAFs by preventing NF-KB driven acquisition of an inflammatory phenotype endowed with angiogenic properties. Moreover, chemotherapy triggers MCL-1 downregulation dependent angiogenic properties in bCAFs, defining MCL-1 as a chemotherapy actionable switch regulating stromal features.

## Results

### Single cell RNA sequencing analysis reveals a transition from myofibroblastic bCAFs to an inflammatory phenotype associated with pro-angiogenic properties after MCL-1 gene silencing

**Fig. 1:**
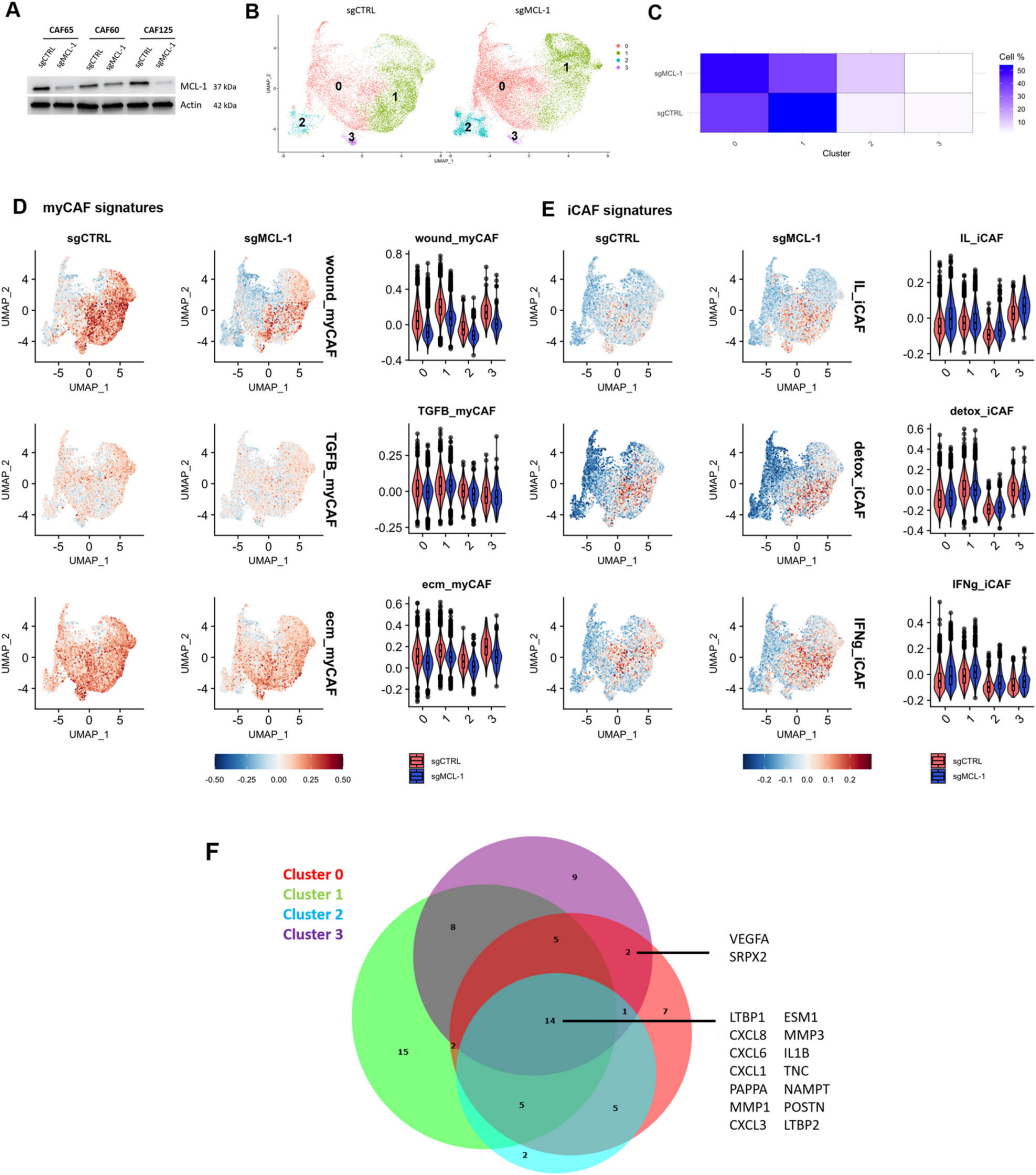
Single cell RNA sequencing analysis reveals a transition from myofibroblastic bCAFs to an inflammatory phenotype associated with pro-angiogenic properties after MCL-1 gene silencing. **A** MCL-1 protein expression level in bCAFs after gene silencing evaluated using western blots. Actin expression was used as loading control (*n* = 3). **B** UMAP showing clusters of bCAF expressing MCL-1 (sgCTRL) or not (sgMCL-1)**. C** Heatmap showing percent of cells in each cluster under all conditions. **D**, **E** (Left) Identity score of **(D)** myCAF signatures (wound-, TGFB- and ECM-myCAF) and (**E)** iCAF signatures (IL-, Detox- and IFNg-iCAF) of Kieffer et al. [7] and (Right) violin plot showing signature score in each cluster in all conditions. **F** Venn diagram of upregulated secreted factors (log2FC > 0.3, *q*-value < 0.01) in each cluster after MCL-1 gene silencinetg in bCAFs built with DeepVenn.

For in depth study of the impact of MCL-1 targeting on the cellular composition of bCAFs, we performed a single cell transcriptomic analysis on three different patient derived primary cultures of bCAFs genetically engineered to be deficient in MCL-1 (bCAFsgMCL-1) or not (bCAFsgCTRL) using CRISPR/Cas9 technology (Fig. 1A). After data integration and unsupervised clustering, we identified four distinct clusters, labeled 0 to 3 (Fig. 1B). Enrichment analysis of gene sets preferentially expressed in each cluster indicated that phenotypic diversity in our primary cultures relied, at least in part, on differences in actors of Rho-GTPase cycle, metabolic process and VEGF-VEGFR signaling (cluster 0/2), cytoplasmic translation (cluster 1), mitotic progression (cluster 2) and protein phosphorylation (cluster 3) (Supplementary Fig. 1A). Importantly, MCL-1 knock down enriched the representation in clusters 0 and 2 at the expense of clusters 1 and 3 (Fig. 1C).

To further decipher how MCL-1 regulates myofibroblastic features [19], we evaluated six bCAF signatures defining myofibroblastic (wound-, TGFB-, ECM-phenotypes) myCAF and inflammatory (IL-, detox-, IFNg-phenotypes) iCAF as determined by Kieffer [7] in each single cell of our landscape. As expected from our culture method, our bCAF primary cultures expressed high levels of myCAF-associated genes. Myofibroblastic features were found across the four different clusters, with varying frequencies. In agreement with our previous study, MCL-1 invalidation reduced myCAF scores. Yet the intensity of decrease differed between phenotypes and clusters. The wound myCAF was particularly sensitive to MCL-1 downregulation, while other myofibroblastic phenotypes somehow maintained in some clusters (TGFb in cluster 1 for instance) (Fig. 1D). The high sensitivity of the wound phenotype is in agreement with its assigned plasticity [8]. Strikingly, MCL-1 silencing also resulted in the acquisition of IL-iCAFs (otherwise rarely found in control bCAFs) in clusters 0 and 3, which showed the most dramatic drop in wound myCAF phenotype (Fig. 1E). Taken together these results argue that loss of MCL-1 in bCAFs promotes a shift from myofibroblastic to inflammatory phenotype in plastic wound myCAFs.

To better characterize the inflammatory and secretory phenotype of bCAF following MCL-1 inhibition, we focused our differential analysis on the expression of genes encoding secreted factors in each cluster. Increased expression of genes encoding pro-inflammatory chemokines/cytokines such as CXCL8, CXCL6, CXCL1, IL1B after MCL-1 gene silencing was found in all clusters (Fig. 1F). Strikingly a significant increase in VEGF-A upon MCL-1 silencing was detected in IL-iCAF-enriched clusters 0 and 3 after MCL-1 inhibition hinting on a possible role for MCL-1 in preventing the pro-angiogenic properties of inflamed CAFs (Fig. 1F and Supplementary Fig. 1B).

### MCL-1 expression in bCAFs is linked to their secretion of VEGF-A

**Fig. 2:**
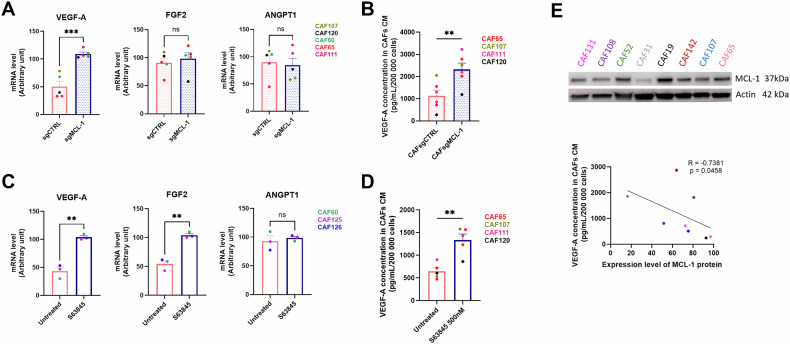
MCL-1 expression in bCAFs is linked to their secretion of VEGF-A. **A** qRT-PCR of VEGF-A, FGF2, and ANGPT1 mRNA in bCAFsgCTRL or sgMCL-1 normalized on RPLPO mRNA expression. Mean and SEM of five independent experiments are represented as relative quantity of mRNA. Student *t*-test, ***P < 0.001, ns not significant. **B** VEGF-A quantification by ELISA in conditioned media (CM) from bCAFs after MCL-1 gene silencing (bCAFsgMCL-1) or not (bCAFsgCTRL). The bCAFs CM were generated during 72 h in EGM2 (Endothelial Cell Growth Medium-2) medium supplemented with 1% of FBS (*n* = 6). Student *t*-test, **P < 0.01. **C** qRT-PCR of VEGF-A, FGF2, and ANGPT1 mRNA in bCAFs treated or not by S63845 500 nM for 18 h normalized on RPLPO mRNA expression. Mean and SEM of three independent experiments are represented as relative quantity of mRNA. Student *t*-test, **P < 0.01, ns non-significant. **D** VEGF-A quantification by ELISA in CM of bCAFs treated or not by S63845 500 nM for 18 h. Results were expressed as concentration (pg/ml) for 200,000 cells (*n* = 5). Student *t*-test, **P < 0.01. **E** Negative correlation (Spearman correlation coefficient *r* = −0.7381; *p* value = 0.0458) between VEGF-A concentration level in bCAFs CM and MCL-1 protein level (relative to actin level) determined by western-blot analysis in 8 primary cultures of bCAFs between passage 2 and 4.

To confirm a role for MCL-1 on the angiogenic features of bCAFs, we first examined the effects of both gene silencing (Fig. 2A, B) and pharmacological inhibition (Fig. 2C, D) of MCL-1 on expression and secretion by bCAFs of different angiogenesis-linked growth factors and cytokines (namely VEGF-A; FGF2, also known as basic-FGF; and ANGPT1). Either MCL-1 gene silencing or inhibition by S63845 [24] resulted in an upregulation of VEGF-A mRNA level (Fig. 2A and C) and in an increase of its secretion by bCAFs (Fig. 2B and D). Pharmacological inhibition of MCL-1 by S63845 also induced FGF2 mRNA level but had no effect on its secretion (data not shown). Notably, we found a significant negative correlation between MCL-1 expression in bCAFs and their VEGF-A secretion (Fig. 2E), suggesting that differences in MCL-1 expression between patient-derived primary bCAFs cultures might account, at least in part, for their differences in angiogenic properties. Targeting BCL-xL, another anti-apoptotic protein expressed by bCAFs [18], by either gene silencing (Supplementary Fig. 2A) or pharmacological inhibition with A1331852 had no detectable effect on pro-angiogenic factors mRNA levels or VEGF-A secretion (Supplementary Fig. 2B–E). Similarly, we found no correlation between the level of BCL-xL expression in bCAFs and their ability to secrete VEGF-A (Supplementary Fig. 2F). These results highlight a specific and unexpected role for the anti-apoptotic protein MCL-1 in the regulation of VEGF-A secretion by bCAFs.

### Targeting of MCL-1 in bCAFs promotes tubulogenesis of endothelial cells in vitro and angiogenesis *in ovo*

**Fig. 3:**
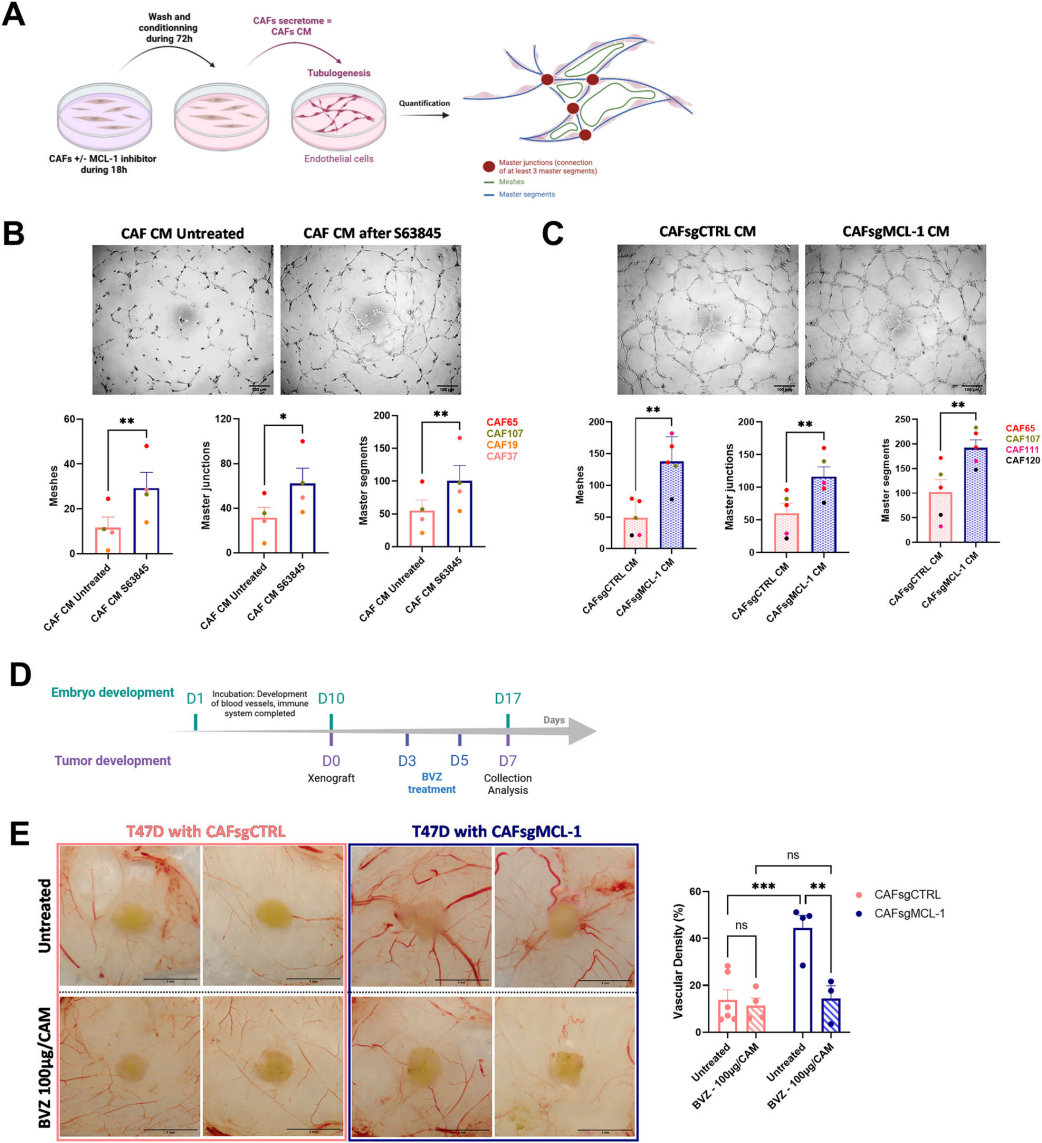
Targeting of MCL-1 in bCAFs promotes tubulogenesis of endothelial cells in vitro and angiogenesis in ovo. **A** bCAFs were treated for 18 h with MCL-1 inhibitor (S63845 500 nM) or not. The treatment was removed and cells were rinsed and cultured for additional 72 h in EGM2 (Endothelial Cell Growth Medium-2) medium supplemented with 1% of FBS. After 72 h, the conditioned media (CM) were applied on HUVECs for tubulogenesis assay. **B**, **C** (Top) Representative images of tubulogenesis assay of HUVECs under (**B**) CM from bCAFs treated or not with MCL-1 inhibitor (S63845 500 nM) or (**C**) CM from bCAFs expressing MCL-1 or not. (Bottom) Quantification of meshes, master junctions, and master segments of endothelial cells under (**B**) CM from bCAFs (untreated and S63845) *n* = 4 or (**C**) CM from bCAFs sgCTRL or sgMCL-1 (*n* = 5). Student *t*-test, *P < 0.5, **P < 0.01. **D** Chronological timeline of CAM model experimentation. After 10 days of embryonic development (ED10), T47D luminal breast cancer cell line with bCAFs expressing or not MCL-1 (bCAFsgCTRL or bCAFsgMCL-1) were xenografted. The tumours were treated with VEGF inhibitor (bevacizumab, BVZ 100 µg/CAM) every 2 days during one week until day 17 of embryonic development (ED17). **E** (Left) Representative pictures of engrafted tumours on CAM at ED17 are shown (Top: untreated or Bottom: treated with bevacizumab, BVZ 100 µg/CAM). (Right) Quantification of blood vessels density around the tumours (within a 5 mm radius of the tumour). Two-way ANOVA, ***P < 0.001, **P < 0.01, ns non-significant.

VEGF-A is a potent pro-angiogenic factor responsible for endothelial cell proliferation, survival, and migration, which promotes angiogenesis during development, tissue vascularization, and cancer progression [25–27]. To evaluate the impact of MCL-1 targeting in bCAFs on angiogenesis, we generated conditioned media during 72 h from bCAFs pre-treated with S63845 (CAF CM after S63845) or not (CAF CM Untreated) for 18 h or from bCAFs silenced for MCL-1 (CAFsgMCL-1 CM) or not (CAFsgCTRL CM). We evaluated the capacities of endothelial cells to organize pseudo-like vessels in vitro with tubulogenesis assay in response to bCAFs secretome (Fig. 3A). Conditioned media from bCAFs pre-treated with S63845 significantly promoted tubulogenesis by inducing more master junctions, master segments, and meshes in comparison with conditioned media from untreated bCAFs (Fig. 3B). In parallel, conditioned media from bCAFs silenced for MCL-1 (CAFsgMCL-1 CM) also significantly enhanced the endothelial tubulogenesis in comparison with conditioned media from the control bCAFs (CAFsgCTRL CM) (Fig. 3C).

We investigated the impact of MCL-1 silencing in bCAFs on the angiogenesis *in ovo* using the chicken chorioallantoic membrane CAM. This vascularized *in ovo* model allows the growth of numerous cell types and is an useful model to assess angiogenesis [28]. In this model, we performed xenografts of tumor models composed of T47D luminal breast cancer cells (chosen for their intrinsically low levels of VEGF-A secretion, approx. 50 pg/mL/200,000 cells, data not shown) mixed with primary bCAFs genetically modified for MCL-1 (CAFsgMCL-1) or not (CAFsgCTRL) (Fig. 3D). Interestingly, one week after xenografts on CAM, we observed that the peritumoral vascular density is significantly increased around the tumors composed of bCAFsgMCL-1 compared to bCAFsgCTRL (Fig. 3E, Top panel). Furthermore, these effects are annihilated with VEGF inhibitor treatment Bevacizumab (BVZ) (Fig. 3E, Bottom panel). These results highlight the strong involvement of MCL-1 in preventing VEGF-A dependant pro-angiogenic effect of bCAFs.

### VEGF-A secretion induced by MCL-1 inhibition in bCAFs involves BAX/BAK activity and is mediated by NF-kB

**Fig. 4:**
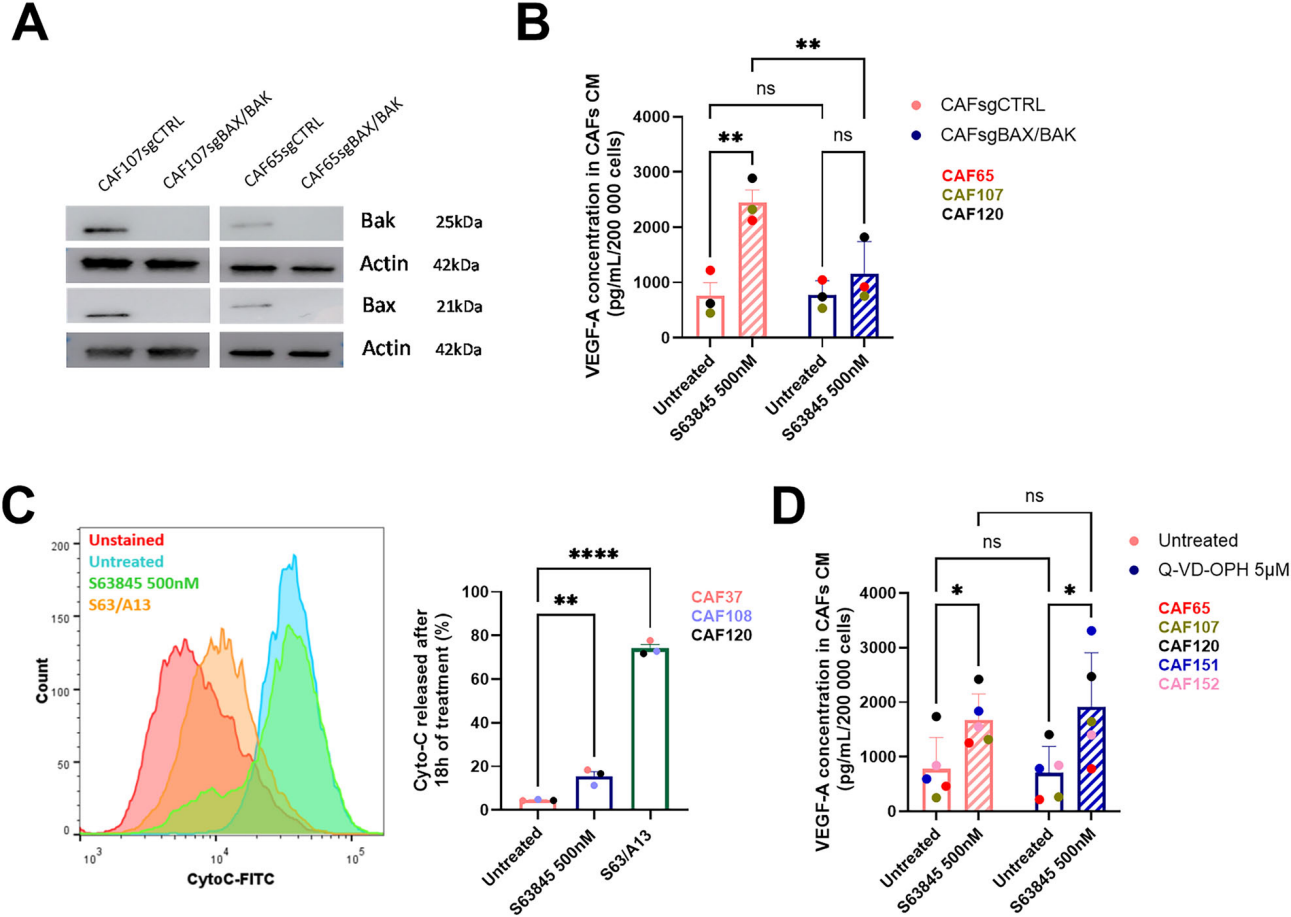
VEGF-A secretion induced by MCL-1 inhibition in bCAFs involves BAX/BAK activity. **A** BAX and BAK protein expression level in bCAFs after gene silencing evaluated using Western blots. Actin expression was used as loading control (*n* = 3). **B** VEGF-A secretion analysed by ELISA in bCAFs CM expressing or not BAX/BAK (bCAFsgBAX/BAK) after 18 h of treatment with MCL-1 inhibitor (S63845 500 nM) or not (*n* = 3). Two-way ANOVA, **P < 0.01; ns non-significant. **C** Cytochrome C released in bCAFs after 18 h of treatment with S63845 (500 nM). Co-treatment with S63845 (500 nM) and A1331852 (BCL-xL inh., 100 nM) served as positive control of Cyto C release (*n* = 3). Student *t*-test, **P < 0.01, ****P < 0.0001. **D** VEGF-A secretion analysed by ELISA in bCAFs CM after 18 h of treatment with MCL-1 inhibitor (S63845 500 nM) in combination with pan-caspase inhibitor (Q-VD-OPH 5 µM) (*n* = 5). Two-way ANOVA, *P < 0.05; ns non-significant.

The canonical anti-apoptotic role of MCL-1 is due to its ability to sequester pro-apoptotic proteins, thereby inhibiting oligomerization of BAX-BAK pro-apoptotic effector proteins and preventing mitochondrial outer membrane permeabilization (MOMP). We assessed the secretion of VEGF-A triggered by S63845 in bCAFs lacking the pro-apoptotic proteins BAX-BAK as a result of a combined CRISPR approach (Fig. 4A). As shown in Fig. 4B, S63845 did not induce VEGF-A secretion by bCAFs devoid of BAX-BAK. Furthermore, inhibition of MCL-1 by S63845 did not trigger overt release of cytochrome C from mitochondria as assessed by flow cytometry (Fig. 4C), in contrast to combined targeting (using for instance S63845 + A1331852). It should also be noted that MCL-1 antagonism did not result in detectable caspase-3/7 activation (Supplementary Fig. 4F, in agreement with our previous work [19]), while the pan-caspase inhibitor Q-VD-OPH did not mitigate S63845 mediated pro-angiogenic effect in bCAFs (Fig. 4D). Altogether these data argue that VEGF-A secretion induced by MCL-1 inhibition in bCAFs involves non-lethal BAX and BAK activity.

**Fig. 5:**
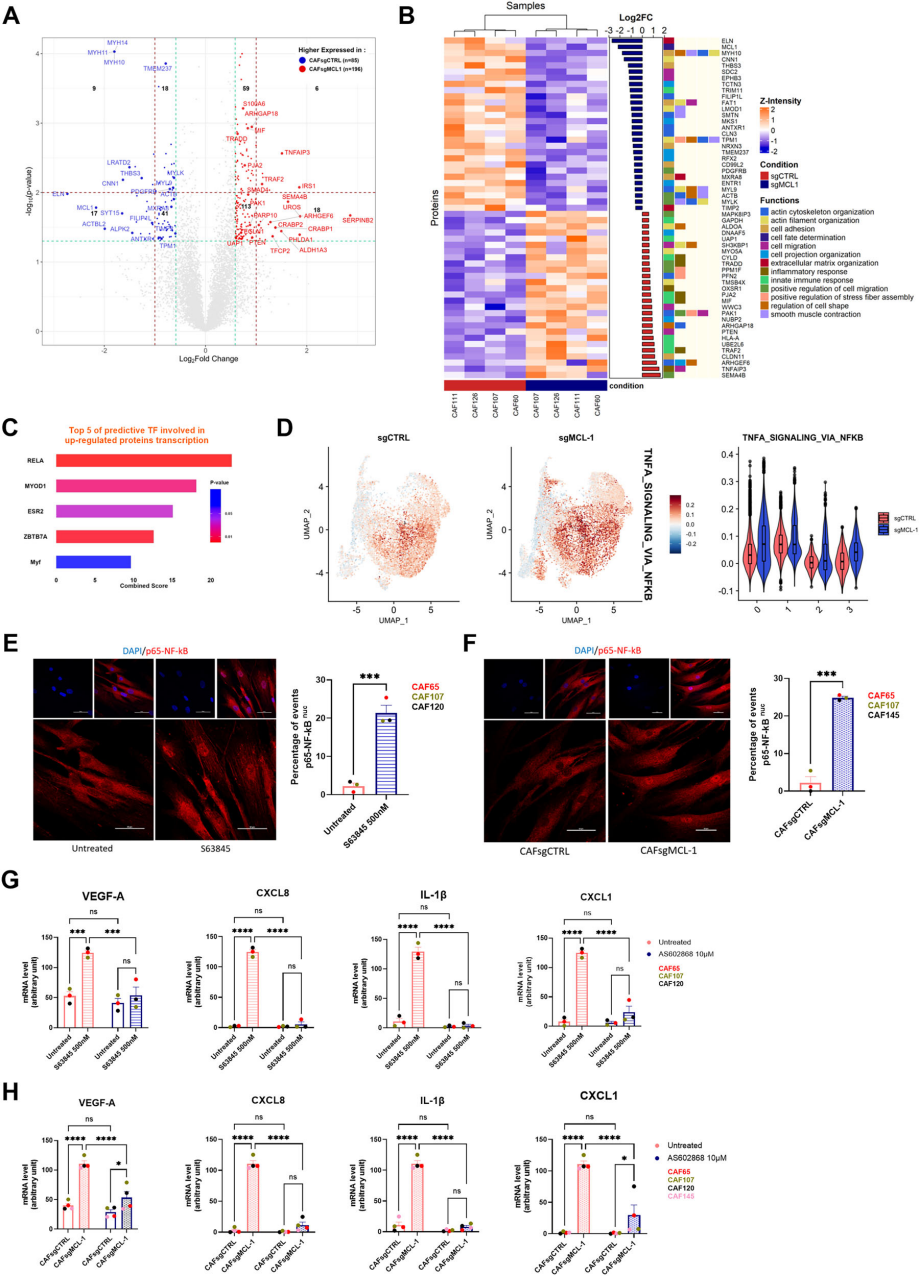
VEGF-A secretion induced by MCL-1 targeting is mediated by NF-kB in bCAFs. **A** Volcano plot showing differential expressed proteins between bCAFs after MCL-1 gene silencing (bCAFsgMCL-1) or not (bCAFsgCTRL) (*n* = 4). The red dots represent the upregulated expressed proteins; the blue dots represent the proteins whose expression is downregulated. **B** A hierarchically clustered heatmap per sample showing differential expressed proteins associated to their biological process (Gene Ontology). Orange and blue represent up and downregulated expression in bCAFs after MCL-1 gene silencing (sgMCL-1) or not (sgCTRL). Color density indicating proteins intensity levels. Log2FC of each protein was indicated on bar plot. **C** Bar plot based on TRANSFAC and JASPAR PWMs databases (Enrichr) of the major transcription factors implicated in the transcription of the upregulated proteins after MCL-1 gene silencing in bCAFs. Combined score = −log(odds.ratio) × *p*-value. **D** (Left) Gene set enrichment analysis with HALLMARK_TNFA_SIGNALING_VIA_NFKB on single cell RNA sequencing data of bCAFs expressing MCL-1 (sgCTRL) or not (sgMCL-1) and (right) violin plot showing signature score in each cluster under all conditions. **E**, **F** Quantification (percentage of cells positive for nuclear p65) (right) and confocal image (left) of p65 staining (red) in (**E**) bCAFs treated or not with S63845 500 nM for 18 h or (**F**) bCAFs sgCTRL or sgMCL-1. The nuclei were counterstained with DAPI (blue) (*n* = 3). Scale bar = 50 μm. Student *t*-test, ***P < 0.001. **G**, **H** qRT-PCR of VEGFA, CXCL8, IL-1β, and CXCL1 mRNA in bCAFs (**G**) after S63845 500 nM for 18 h (*n* = 3) or (**H**) sgCTRL and sgMCL1 (*n* = 4), in presence or not with an IKKβ inhibitor (AS602868, 10 µM) normalized on RPLPO mRNA expression. Two-way ANOVA, ****P < 0.0001, ***P < 0.001, ns non-significant.

To circumvent the phenotypic effect of targeting MCL-1 in bCAFs we performed a global proteomic analysis by mass spectrometry on four different primary cultures of bCAFs knock down for MCL-1 or not. We identified a total of 8184 proteins including 85 downregulated proteins and 196 upregulated proteins (log2FC > 0.58 and *p*-value < 0.05) after MCL-1 silencing in bCAFs (Fig. 5A). Gene Ontology (GO) pathway enrichment analysis confirmed that MCL-1 gene silencing in bCAFs induced a decreased expression of proteins associated with their myofibroblastic phenotype and an increased expression of proteins implicated in inflammatory and innate immune response. These included TRADD, TRAF2, MIF, TNFAIP3, and CYLD related to the transcription factor NF-kB activity (Fig. 5B). Moreover, TRANSFAC and JASPAR PWMs databases explorations indicated that a majority of upregulated proteins after MCL-1 gene silencing are transcriptional targets of RELA/NF-kB, which contributes to inflammatory phenotype acquisition (Fig. 5C). This corroborates our single cell RNA sequencing analysis, as NF-kB-regulated genes are increased in clusters 0 and 3 after MCL-1 gene silencing (Fig. 5D). MCL-1 targeting with S63845 or silencing by CRISPR-Cas9 induced a significant increase of NF-kB nuclear translocation in bCAFs (Fig. 5E, F) and NF-kB activity blockade with an IKKβ inhibitor (AS602868) counteracted VEGF-A mRNA level increase after MCL-1 targeting as well as the increase in CXCL8, IL-1β and CXCL1 mRNA (Fig. 5G, H). These results establish the implication of NF-kB signaling in BAX/BAK-dependent effects of MCL-1 inhibition on VEGF-A synthesis. Of note, we detected no effect of STING or TBK1 inhibition in bCAFs on the NF-kB-dependent pro-inflammatory effects of MCL-1 targeting, ruling out the involvement of cGAS-STING/TBK1-dependent NF-kB activation downstream of BAX-BAK mitochondrial permeabilization [29] in this process (Supplementary Fig. 3A–E).

### Chemotherapy modulates the pro-angiogenic properties of bCAFs through NOXA-mediated MCL-1 degradation and subsequent activation of NF-κB signaling

**Fig. 6:**
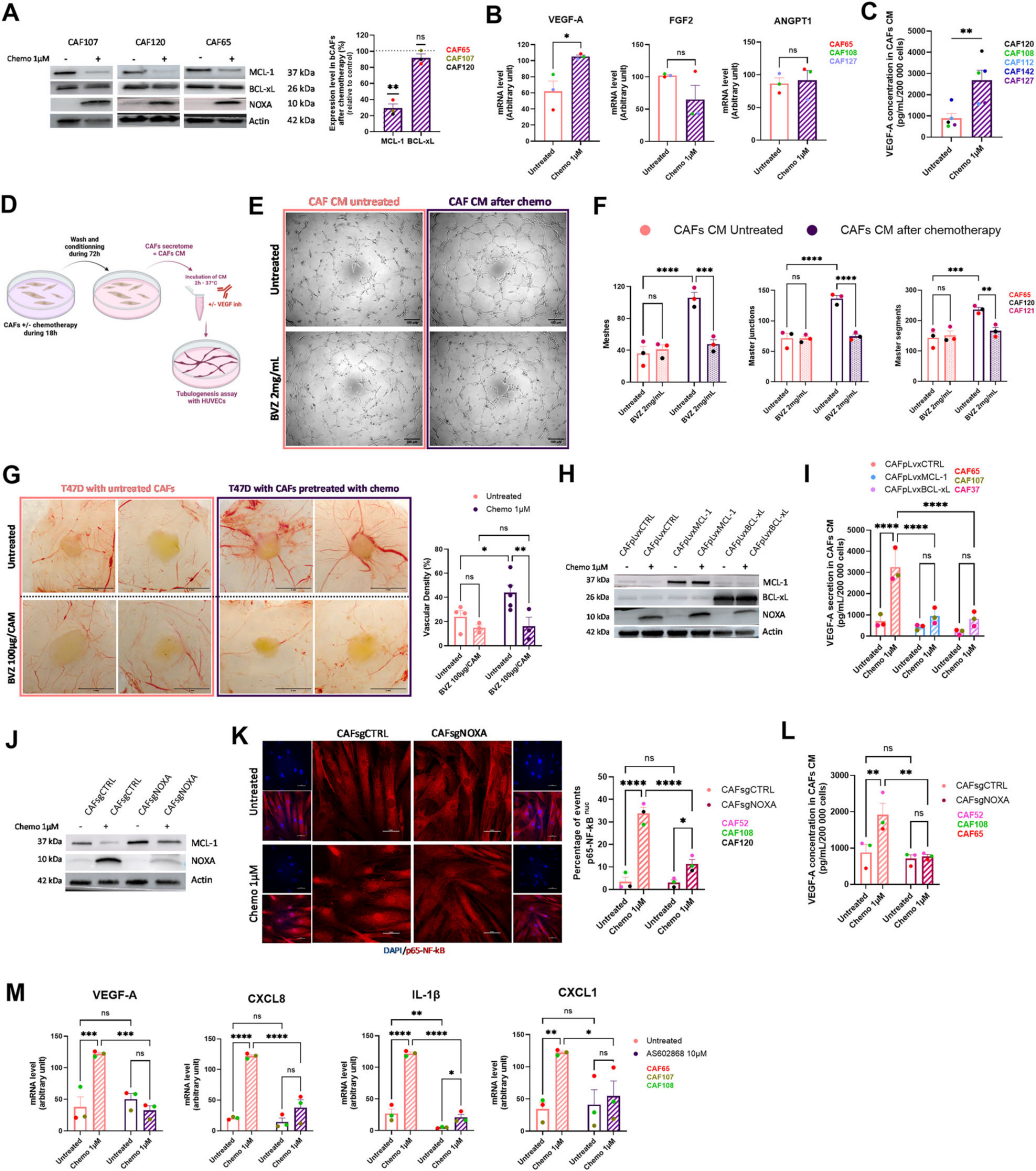
Chemotherapy modulates the pro-angiogenic properties of bCAFs through NOXA-mediated MCL-1 degradation and subsequent activation of NF-κB signaling. **A** (Left) Representative experiments of proteins expression level (MCL-1, BCL-xL, and NOXA) in bCAFs after 18 h of chemotherapy treatment (5-Fluorouracil 22 µM, Cisplatin 11 µM, and Doxorubicin 1 µM) or not evaluated using western blots, Actin expression was used as loading control. (Right) Quantification of the amounts of MCL-1 and BCL-xL protein band relative to Actin in bCAFs after chemotherapy treatment (*n* = 3), results are expressed as a ratio relative to untreated bCAFs. One sample *t*-test, **P < 0.01, ns non-significant. **B** RT-qPCR of VEGF-A, FGF2, and ANGPT1 mRNA in bCAFs after 18 h of chemotherapy treatment or not normalized on RPLPO mRNA expression. Mean and SEM of three independent experiments are represented as relative quantity of mRNA. Student *t*-test, *P < 0.05, ns not significant. **C** VEGF-A was analysed by ELISA in bCAFs CM after 18 h of chemotherapy. Student *t*-test, **P < 0.01. **D** bCAFs were treated or not with chemotherapy for 18 h. The treatment was removed and cells were rinsed and cultured for additional 72 h in EGM2 medium supplemented with 1% of FBS. After 72 h, conditioned media (CM) were recovered, and a portion was incubated with VEGF inhibitor (BVZ, 2 mg/mL) for 2 h −37 °C before adding on HUVECs for tubulogenesis assay. **E** Representative images of tubulogenesis assay of HUVECs cultured in conditioned media from bCAFs treated with chemotherapy or not +/− BVZ (2 mg/mL). **F** Quantification of meshes, master junctions, and master segments of HUVECs cultured in conditioned media from bCAFs treated or not with chemotherapy +/− BVZ (2 mg/mL) (*n* = 3). Two-way ANOVA, **P < 0.01, ***P < 0.001, ****P < 0.0001, ns non-significant. **G** (Left) Representative pictures of engrafted tumours on CAM at ED17, tumours are composed of T47D cells and bCAFs pre-treated or not with chemotherapy for 18 h before xenograft (Top). The tumours were treated with VEGF inhibitor (bevacizumab, BVZ 100 µg/CAM) every 2 days during one week until ED17 (Bottom). (Right) Quantification of blood vessels density around the tumours (within a 5 mm radius of the tumour). Two-way ANOVA, **P < 0.01, *P < 0.05, ns non-significant. **H** MCL-1, BCL-xL, and NOXA proteins expression levels in bCAFs, surexpressing MCL-1 (CAFpLvxMCL-1) or BCL-xL (CAFpLvxBCL-xL) or not (CAFpLvxCTRL) after 18 h of chemotherapy were evaluated using Western blots analysis. Actin expression was used as loading control. **I** VEGF-A secretion was analysed by ELISA in bCAFs CM in the same conditions as (**H**) (*n* = 3) Two-way ANOVA, ****P < 0.0001; ns non-significant. **J** MCL-1 and NOXA proteins expression levels in bCAFs expressing NOXA (CAFsgCTRL) or not (CAFsgNOXA) after 18 h of chemotherapy were evaluated using Western blots analysis. Actin expression was used as loading control. **K** Quantification (percentage of cells positive for nuclear p65) (right) and confocal image (left) of p65 staining (red) in bCAFs expressing NOXA (CAFsgCTRL) or not (CAFsgNOXA) and treated with chemotherapy (1 µM) for 18 h or not. The nuclei were counterstained with DAPI (blue) (*n* = 3). Scale bar = 50 μm. Two-way ANOVA, ****P < 0.0001, *P < 0.05, ns not significant. **L** VEGF-A secretion was analysed by ELISA in bCAFs CM in the same conditions as (**J**). **M** qRT-PCR of VEGFA, CXCL8, IL-1β, and CXCL1 mRNA in bCAFs treated with chemotherapy (1 µM) for 18 h or not in combination with an IKKβ inhibitor (AS602868, 10 µM) or not normalized on RPLPO mRNA expression (*n* = 3). Two-way ANOVA, ****P < 0.0001, ***P < 0.001, **P < 0.01, *P < 0.05, ns non-significant.

We finally analysed whether the function exerted by MCL-1 in bCAFs, described above, is modulated by chemotherapy. One standard of breast cancer treatment involves the combination of three chemotherapies: anthracycline (like doxorubicin), alkylating agents (like cisplatin), and anti-metabolites (like 5-fluorouracil). Under our conditions, chemotherapy-induced less than 15% cell death in CAFs after 18 h of treatment and we did not observe any additional cell death during the following 72 h of medium conditioning (Supplementary Fig. 4A–C). In contrast, it drastically reduced MCL-1 protein levels while simultaneously increasing the pro-apoptotic NOXA protein in bCAFs (Fig. 6A). Indeed, in many cell types, DNA-damaging chemotherapeutic agents stimulate transcription of NOXA [30] an endogenous inhibitor of MCL-1, that can interact with it to promote its degradation [31–33]. BCL-xL level remained unchanged in all conditions (Fig. 6A).

We found that MCL-1 protein level decrease by chemotherapeutic drugs was accompanied by an increase of VEGF-A mRNA without modification of FGF2 and ANGPT1 mRNA level in bCAFs (Fig. 6B). VEGF-A secretion was increased by bCAFs after chemotherapy and accordingly, conditioned media from bCAFs in this condition promoted endothelial tubulogenesis in a BVZ sensitive manner (Fig. 6C–F). Indeed, *in ovo*, we demonstrated that bCAFs pre-treated with chemotherapy before xenograft on CAM induced more blood vessels around the tumor compared to xenograft with bCAFs untreated (Fig. 6G). Our results highlight the impact of chemotherapy on pro-angiogenic phenotype of bCAFs. We demonstrated that MCL-1 overexpression in bCAFs (Fig. 6H and Supplementary Fig. 4D) mitigates the chemotherapy-induced increase in VEGF-A secretion (Fig. 6I). A similar effect was observed with BCL-xL overexpression, suggesting that antagonizing BAX/BAK is critical for chemotherapeutic stress to modulate VEGF-A secretion by bCAFs (Fig. 6I). Given that the pro-apoptotic protein NOXA is an inhibitor of MCL-1 and is markedly upregulated in response to chemotherapy (Fig. 6A), we further investigated the contribution of the endogenous NOXA/MCL-1 axis to chemotherapy-induced pro-angiogenic effects by generating bCAFs silenced for NOXA (Supplementary Fig. 4E). NOXA depletion preserved MCL-1 expression, inhibited the nuclear translocation of p65-NF-κB, and attenuated VEGF-A secretion in response to chemotherapy (Fig. 6J–L). Moreover, pharmacological inhibition of NF-κB activation using the IKK inhibitor AS602868 significantly reduced the chemotherapy-enhanced expression of pro-angiogenic and pro-inflammatory mediators, including VEGF-A, CXCL8, IL-1β, and CXCL1 at the mRNA level in bCAFs (Fig. 6M). Of note and, consistent with the absence of cell death and caspase 3 cleavage induced by chemotherapy in our conditions (Supplementary Fig. 4F), chemotherapy-induced VEGF-A secretion was not detectably affected by co-treatment with a caspase inhibitor, Q-VD-OPH (Supplementary Fig. 4G).

**Fig. 7:**
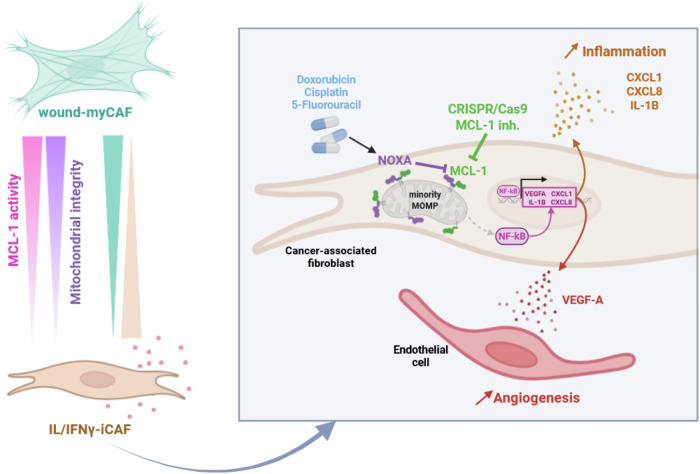
The pro-angiogenic phenotype of bCAFs is dependent on MCL-1 activity. Graphical abstract: Targeting MCL-1 induces BAX/BAK-dependent mitochondrial dysfunction, which leads to NF-κB nuclear translocation and VEGF-A transcription and secretion, ultimately promoting angiogenesis. Additionally, chemotherapy enhances this pro-angiogenic phenotype through a mechanism involving the NOXA/MCL-1/NF-κB axis.

Collectively, these findings highlight an unexpected role of MCL-1 in the phenotypic plasticity of bCAFs and support a model in which chemotherapy promotes a pro-inflammatory and pro-angiogenic phenotype in bCAFs through increased NOXA expression, NOXA-mediated MCL-1 degradation, and subsequent activation of NF-κB signaling (Fig. 7).

## Discussion

In this study, we highlight a phenotypic transition of bCAFs from a myofibroblastic to an inflammatory and pro-angiogenic phenotype and show that it is controlled by MCL-1 canonical activity. This emphasizes the role of MCL-1 in the overall organization of breast cancer ecosystems, in which it overexpression has frequently been associated with resistance to treatment [18], [34]. This also puts forth a plasticity in bCAFs populations that could explain their phenotypic response to chemotherapy.

In breast cancer, two major pro-tumoral bCAFs subpopulations have been described, including myCAFs associated with contractile and immunosuppressive phenotype and iCAFs known for their extensive production of inflammatory factors [7]. We have previously shown that targeting the anti-apoptotic protein MCL-1 in bCAFs reduced their contractile and invasive capacities. Studies in cardiomyocytes reported similar findings, suggesting a role for MCL-1 in contractile cell function maintenance [35]. Our single-cell RNA sequencing analysis of primary bCAF cultures following MCL-1 gene silencing demonstrates that the myofibroblastic wound-myCAF phenotype of bCAFs is highly dependent on MCL-1. Moreover, the loss of MCL-1 expression induces a phenotypic shift towards an inflammatory profile, particularly characterized by the acquisition of an IL-iCAF phenotype, incidentally underscoring the high plasticity of bulk bCAFs populations, even in vitro. In their recent study, Croizer et al. [8] identified a differentiation pathway among bCAF subpopulations, with Detox-iCAF proposed as the origin of all other bCAF subtypes. In our work, wound-myCAFs were most affected by MCL-1 gene silencing, somehow consistent with their higher phenotypic plasticity compared to ECM-myCAFs and TGFβ-myCAFs that are at more advanced differentiation stages. Since MCL-1 knock down favors an IL-iCAF phenotype while exerting no detectable effect on a Detox-iCAF one, we propose that a novel MCL-1-dependent reversible differentiation pathway leading to the transition from IL-iCAFs to Wound-myCAFs exists. This is in agreement with Croizer’s study showing a major role for YAP1 transcription factor activity in the maintenance of the myCAFs phenotype, as we showed that inhibition of MCL-1 in bCAFs leads to cytoplasmic retention of YAP1 [8]. In prostate cancer, YAP1 has also been shown to act as a guardian of the myCAF phenotype by limiting the iCAF phenotype via inhibition of NF-κB activity [36]. Acquisition of inflammatory/angiogenic features in plastic bCAFs upon MCL-1 invalidation might equally result from or cause the loss of (wound) myofibroblastic observed. However, MCL-1 appears to differentially regulate these two phenotypes at the molecular level. We showed that the loss of myofibroblast phenotype induced by MCL-1 inhibition (characterizing the first step in the transition from Wound-myCAFs to IL-iCAFs) was associated with mitochondrial fragmentation in a DRP1-dependent manner, yet without the need for pore-forming effectors of the MOMP, BAX, and BAK. In contrast, we herein showed that inhibition of MCL-1 leading to an IL-iCAF (secreting VEGF-A) phenotype required BAX/BAK activity. While this happens without overt cytochrome c release or caspase activation, triggering cell death ([19] and supplementary data), it nevertheless argues that MCL-1 prevents the acquisition of an inflammatory/angiogenic phenotype by bCAFs as a canonical guardian of their mitochondrial integrity. We thus propose that complete transition from Wound-myCAFs to IL-iCAFs upon MCL-1 invalidation comprise two molecular events at the mitochondrial level (mitochondrial fragmentation and permeabilization, respectively).

Our findings establish a connection between MCL-1 inhibition, NF-κB activation, and the acquisition of an inflammatory and pro-angiogenic phenotype by bCAFs. During MOMP, released mitochondrial contents like mtDNA can trigger inflammation via the cGAS-STING-NF-κB pathway [29]. Our study demonstrated that S63845-induced transcription of VEGF-A, CXCL8, IL-1β, and CXCL1 in bCAFs requires NF-κB activity but appears independent from STING/TBK1. Evidence suggests NF-κB activation can occur through NEMO (IKKγ) recruitment to ubiquitinylated mitochondria post-MOMP, forming a complex with IKKβ and IKKα, leading to IκBα degradation and NF-κB nuclear translocation [22, 23]. Further investigation is needed to clarify NF-κB activation mechanisms after S63845-induced non-lethal BAX/BAK activation in bCAFs.

Our definition of MCL-1 as a key molecular switch between the opposing myofibroblastic and inflammatory phenotypes within bCAFs populations increase understanding of the effects of chemotherapy on bCAFs, which we show here (in the form of a doxorubicin, cisplatin, 5-fluorouracil treatment) to downregulate MCL-1 expression. The latter was shown to be downregulated by anthracyclines, which are part of the chemotherapeutic regimen used in the treatment of breast cancer. We infer that chemotherapy-induced DNA damage and transcriptional arrest in bCAF leads to a decrease in MCL-1 levels, further amplified by degradation due to increase NOXA levels. Importantly, we established that chemotherapy promotes VEGF-A secretion by bCAFs (which contributes to endothelial cell tubulogenesis in vitro and angiogenesis *in ovo)* as well as the acquisition of an inflammatory phenotype by bCAFs. This is mitigated by overexpression of either MCL-1 or BCL-xL in agreement with the notion that BAX/BAK inhibition is involved. Importantly, this is also abrogated by NOXA silencing, which counteracts MCL-1 degradation, defining endogenous NOXA and MCL-1 as key regulators of CAFs phenotypic changes in response to chemotherapy. It is relevant to note here that prior reports have described MCL-1 as an inhibitor of chemotherapy-induced senescence across various models [37, 38] and that chemotherapy induces in some cell types a senescence-associated secretory phenotype which includes pro-angiogenic factors [39, 40]. However, in our experimental conditions, senescence does not appear to contribute to the acquisition of the pro-angiogenic phenotype following MCL-1 antagonism (data not shown). Our study is nevertheless in line with these findings, as it highlights a critical role for MCL-1 in restraining the emergence of an inflammatory/pro-angiogenic phenotype in bCAFs in response to chemotherapy [37, 38]. The role of this protein in balancing the inflammatory and angiogenic versus myofibroblastic features of bCAFs underlines possible adverse effects of its modulation by chemotherapy on the stroma. In fact, at clinical level, micro-vessel density is correlated with a poor prognosis with greater likelihood of metastatic disease and shorter survival rate for breast cancer patient [41–43]. These results underscore the importance of further investigating the predictive significance of stromal MCL-1 expression within well-defined clinical settings.

## Methods and materials

### Cell culture and reagents

Fresh human mammary samples were obtained from treatment-naive patients with invasive carcinoma after surgical resection at the Institut de Cancérologie de l’Ouest, Nantes/Angers, France. As required by the French Committee for the Protection of Human Subjects, informed consent was obtained from enrolled patients and protocol was approved by Ministère de la Recherche (agreement no.: DC-2012-1598) and by local ethic committee (agreement no.: CB 2012/06). To isolate bCAFs from fresh samples, breast tissues were cut into small pieces in Dulbecco’s Modified Eagle Medium (DMEM Thermo Fisher Scientific) supplemented with 10% FBS, 2 mM glutamine, and 1% penicillin/streptomycin and placed in a plastic dish. bCAFs were isolated by their ability to adhere to plastic. After isolation, the fibroblasts were cultured in the same medium. Fibroblasts were used in the experiments before the ninth passage.

For the CRISPR Cas9-induced knock-out (KO) primary bCAFs, singleguide (sg) RNA sequences targeting human genes were designed using the CRISPR design tool (http://crispor.tefor.net). The following guide sequences were cloned in the lentiCRISPRV2 vector that was a gift from Feng Zhang (Addgene plasmid #52961): sgMCL-1: 5′-CTGGAGACCTTACGACGGGT-3′, sgBCL-xL: 5′-GCAGACAGCCCCGCGGTGAA-3′, sgBAK: 5′-GCCATGCTGGTAGACGTGTA-3′, sgBAX: 5′-AGTAGAAAAGGGCGACAACC-3′, sgSTING: 5′-GCAGGCACTCAGCAGAACCA-3′, and sgNOXA 5′-TCGAGTGTGCTACTCAACTC-3′. MCL-1 and BCL-xL overexpressing bCAFs were established by viral infection with retroviruses containing vector coding for MCL-1 or BCL-xL (pLVX-EF1alpha-IRES-Puro). Empty vector was used as control. Cells were selected using 1 μg/ml puromycin and protein extinction were confirmed by immunoblot analysis.

The human breast cancer cell lines T47D were purchased from American Type Culture Collection (Bethesda, MD, USA) and was cultured in RPMI medium supplemented with 10% FBS and 2 mM glutamine.

Human umbilical vein endothelial cells (HUVECs) were freshly isolated from umbilical cords obtained from Nantes Hospital Maternity from different donors with informed consent from all subjects, as previously described [44]. Endothelial cells (ECs) were routinely cultured in Endothelial basal medium (EGM2; containing 2% fetal bovine serum (FBS); PromoCell, Heidelberg, Germany) supplemented with Endothelial growth medium supplement pack (ECGM-2; PromoCell, Heidelberg, Germany) and 100 IU/mL penicillin and 100 mg/mL streptomycin on 0,1% gelatin-coated flasks. Cells were used from passage 1 to 6. All cells were cultured at 37 °C and 5% CO_2_.

### Treatments

Doxorubicin (Selleckhem #S1208), Cisplatin (Selleckhem #S1166), and 5-Fluorouracil (Selleckhem #S1209) were used alone or mixed at 1, 11, and 22 μM, respectively to obtain chemotherapy treatment. A1331852 (MedChemExpress #HY-19741), S63845 (ChemieTek #CT-S63845), Q-VD-OPH (R&D Systems, Abingdon, UK), VEGF inhibitor (Bevacizumab, Aybintio, Samsung Bioepis), ProtacTBK1 (Biotechne, 7259), ProtacCTRL (TBK1 Control Protac, Biotechne, 7260), and AS602868 (Merck-Serono International SA) were used at indicated concentrations.

bCAFs are still treated for 18 h in DMEM containing 1% FBS. For TBK1 degradation experiments, S63845 was added for 18 h after a 3 h incubation with Protac. For experiments with pan-caspase inhibitor, QVDOPH was used for 18 h in combination or not with S63845 or chemotherapy. QVDOPH was also added during the 72 h of conditioning in EGM2.

### Single-cell RNA sequencing

Single cell RNA sequencing was performed using the Chromium Single-Cell 3′ v3.1 kit from 10× Genomics, following the manufacturer’s protocol. The libraries were sequenced on the NovaSeq 6000 platform (Paired-end, with 28 bp for Read1 and 90 bp for Read2). The raw BCL files were demultiplexing and aligned to the reference genome (refdata-cellranger-GRCh38-3.0.0) using the Cell Ranger Software Suite (v.7.0.1).

### Preprocessing and quality control of single-cell RNA sequencing data

The raw data were extracted from 10× format files using the *Read10X* function from the Seurat package (v4.4.0) in R v.4.1.1. Quality control filtering was applied to the feature-barcode gene expression matrix to retain only high-quality cells. Cells were selected based on the following criteria: more than 5000 unique molecular identifiers, more than 2000 detected genes, and less than 20% of reads mapped to mitochondrial genes to exclude potentially dead or dying cells. Doublets were detected using the scDblFinder R package (v.1.6.0). A score was computed for each cell, and doublets were identified by applying the threshold defined by the scDblFinder package. Detailed quality control metrics are provided in Supplementary Table 1.

### Single-cell RNA sequencing analysis

Data normalization for each dataset was performed using the *NormalizeData* function from the Seurat package. Variable features were identified using the vst method, selecting the 2000 most highly variable genes per dataset. To integrate the datasets, features that were repeatedly variable across all datasets were selected using the *SelectIntegrationFeatures* function. Data were then scaled using the ScaleData function, and cell cycle effects were minimized by regressing out S and G2M phase scores. To perform dataset integration, anchors were identified using the *FindIntegrationAnchors* function, followed by integration of the data with the *IntegrateData* function, creating an integrated assay.

Principal Component Analysis (PCA) was performed using the *RunPCA* function, and Louvain graph-based clustering was applied to the first 30 principal components. The resulting data were visualized using Uniform Manifold Approximation and Projection (UMAP) with the *RunUMAP* function. Distinct clusters were identified using the *FindNeighbors* and *FindClusters* functions, with a resolution of 0.1.

For each defined cluster, gene marker analysis was conducted using the *FindAllMarkers* function with the following parameters: only.pos = TRUE, min.pct = TRUE, and logfc.threshold = 0.25. Finally, signature scores were calculated using the *AddModuleScore* function.

For pathway enrichment analysis, we used fgsea package (v1.33.1), marker genes were identified using the FindAllMarkers function with parameters min.pct = 0 and logfc.threshold = 0. Genes were then ranked according to their differences in gene expression to the other clusters. Enrichment was assessed using a set of pathways that were selected by analysis of genes preferentially expressed in each cluster.

The list of genes encoding secreted factors was based on the UniProtKB list of proteins located outside cell membranes (Cellular component—Secreted). A manual curation process, based on literature, was performed to remove certain genes not coding for extracellular proteins.

### Proteomic analysis by LC-MS/MS

The pelleted cells were quickly thawed and proteins were concomitantly extracted, denatured and thiol groups were chemically reduced in 200 µL of 0.1% Rapigest-SF® (Waters), 5 mM DTT and 50 mM ammonium bicarbonate pH 8.5, in a thermomix set at 95 °C for 30 min under 750 rpm shaking. Sonication was performed twice 30 s using an ultrasonic processor probe sonicator (130 W, 20 KHz) at 20% power (Thermo Fisher Scientific). Alkylation of free thiols was performed by adding S-Methyl methanethiosulfonate (10 mM final concentration from Sigma) with a further ten-minute incubation at 37 °C and 750 rpm shaking. 5 µg porcine trypsin (Sciex) was used to generate bottom-up LC MS-compatible peptides, and incubated at 37 °C overnight. Resulting peptides were then cleared from potential insoluble material by centrifugation at 16000 × *g* for 15 min. The supernatant-containing peptides was desalted using in-house pipette tips-packed with C18, described as “Stage-tips” procedure [45]. Peptides were then eluted and dried in a vacuum centrifuge concentrator (Savant SPD121P from Thermo), resuspended in 42 µL of 0.1% Formic Acid (FA) and submitted to microBCA peptide assay (Thermo) to allow the injection of 200 ng of peptides to be injected in LC MSMS in DIA-PASEF mode as follows: 2 µL of each sample was loaded and separated on a C18 reverse phase column (Aurora series 1.6 µm particles size, 75 µm inner diameter and 25 cm length from IonOptics, Fitzroy Australia) using a NanoElute LC system (Bruker, Bremen Germany). Eluates were electrosprayed into a timsTOF Pro 2 mass spectrometer (Bruker) for the 60 min duration of the hydrophobicity gradient ranging from 99% of solvent A containing 0.1% FA in milliQ-grade H2O to 27% of solvent B containing 80% ACN plus 0.1% FA in mQ-H2O. The mass spectrometer acquired data throughout the elution process and operated in data-independent analysis (DIA) with PASEF-enabled method using the TIMS-Control software (Bruker). Samples were injected in batch replicate order to circumvent possible interferences of technical biases.

The m/z acquisition range was 250–1201 with an ion mobility range of 0.7–1.25 1/K0 [V s/cm^2^], which corresponded to an estimated cycle time of one second. DIA-PASEF windows, and collision energy were also left to default with a base of 0.85 1/K0 [V s/cm^2^] set at 20 eV and 1.30 1/K0 [V s/cm^2^] set at 59 eV. TOF mass calibration and TIMS ion mobility 1/K0 were performed linearly using three reference ions at 622, 922, and 1222 m/z (Agilent Technology, Santa Clara, CA, USA).

### LC-MS/MS raw data analysis

The raw data were extracted, normalized, and analyzed using Spectronaut® 18.0.230605.50606 (Biognosys) in DirectDIA+ mode, which modelized elution behavior, mobility and MS/MS events based on the Uniprot/Swissprot sequence 2020 database of 20,365 human proteins and 381 entries of common contaminant proteins. Protein identification false discovery rate was restricted to 1% maximum, with a match between runs option enabled, and inter-injection data normalization. The enzyme’s specificity was trypsin’s. The precursor and fragment mass tolerances were set to 15 ppm. Acetyl (Protein N-term) and oxidation of methionines were set as variable modifications, while thiol groups from cysteines were considered completely alkylated by methylation. A minimum of two ratios of peptides was required for relative quantification between groups. Protein quantification analysis was performed using Label-Free Quantification (LFQ) intensities. Spectronaut statistical tools were used to visualize data, assess quality, and quantitatively compare datasets. The resulting proteins LFQ values were log2(1.5) transformed and importantly a minimum of 5% of available *p*-values in at least one group was needed to include proteins in the differential abundance analysis. The proteomic analysis was conducted using a custom-developed R script (v.4.4.1). Gene Ontology (GO) terms related to “Biological Process” were assigned using the Ensembl database via the biomaRt R package (v.1.0.7) (https://cran.r-project.org/web/packages/biomartr/index.html). Duplicate proteins were then filtered by retaining only the gene with the lowest *p*-value and we considered that a protein was expressed when it was present in at least 3 out of 4 samples in each condition. A manual curation process, informed by a thorough review of relevant literature, was performed to assign some proteins to relevant Biological Process categories. Volcano plot was generated using the ggplot2 package (v.3.5.1), while heatmap was built with ComplexHeatmap package (v.2.20.0) (https://bioconductor.org/packages/release/bioc/html/ComplexHeatmap.html) using Euclidean distance and ward D2 clustering method.

### RNA isolation and quantitative real-time PCR

Total RNA was isolated using Nucleospin RNA (Macherey-Nagel, Hoerdt, France) and transcribed into cDNA by Maxima First Strand cDNA synthesis Kit (Thermo Scientific). Quantitative RT-PCR (qPCR) was performed using the EurobioGreen qPCR Mix Lo-Rox with qTOWER (Analityk-Jena, Jena, Germany). Reaction was done in 10 μl final with 4 ng RNA equivalent of cDNA and 150 nM primers. Relative quantity of mRNA was estimated by Pfaffl method [46] and normalized on the average relative quantity of one housekeeping gene.

RPLP0 5′-AACCCAGCTCTGGAGAAACT/CCCCTGGAGATTTTAGTGGT-3′

GAPDH 5′-CAAAAGGGTCATCATCTCTGC/AGTTGTCATGGATGACCTTGG-3′

FGF2 5′-CTTCCTGCGCATCCACCCCG/AGCCAGGTAACGGTTAGCACACA-3′

ANGPT1 5′-GCTCCACACGTGGAACCGGA/CCAGCATGGTAGCCGTGTGGT-3′

VEGF-A 5′-AAGGAGGAGGGCAGAATCAT/CCAGGGTCTCGATTGGATGG-3′

IL-1β 5′-TGGCAATGAGGATGACTTGT/GGAAAGAAGGTGCTCAGGTC-3′

CXCL8 5′-AAGCTGGCCGTGGCTCTCTTG/TTCTGTGTTGGCGCAGTGTGGT-3′

CXCL1 5′-AACAGCCACCAGTGAGCTTC/GAAAGCTTGCCTCAATCCTG-3′

### Immunoblot analysis

Cells were re-suspended in lysis buffer (1% SDS; 10 mM EDTA; 50 mM TrisHcl pH 8.1; 1 mM PMSF; 10 μg/ml aprotinin; 10 μg/ml leupeptin; 10 μg/ml pepstatin; 1 mM Na3VO4 and 50 mM NaF). For Western blotting, following SDS–PAGE, proteins were transferred to 0.45 µM nitrocellulose membranes using Trans-Blot® Turbo™ Transfer System Cell system (Bio-Rad). The membrane was then blocked in 5% nonfat dry milk, TBS 0.05% Tween 20 and incubated with primary antibody overnight at 4 °C. Blots were incubated with the appropriate secondary antibodies for 1 h at room temperature and visualized using the Fusion FX (Vilber). The used primary antibodies were anti-MCL-1 (Cell Signaling, 94296, 1/1000), anti-Bax (Cell Signaling, 5023, 1/1000), anti-Bak (Cell signaling, 1210, 1/1000), anti-BCL-xL (Cell Signaling, 2764, 1/1000), anti-NOXA (Abcam, ab13654, 1/500), TBK1 (Cell Signaling, 3504, 1/1000), STING (Cell Signaling, 13647, 1/1000), and anti-β-actin (Millipore, MAB1501R, 1/2000).

### ELISA

For the preparation of conditioned media (CM), 170,000 bCAFs were seeded in one well of a 6-well plate and were treated as indicated during 18 h in DMEM 1% FBS. The cells are washed with PBS and cultured in EGM2 supplemented with 1% FBS for additional 72 h. CM were then collected and centrifuged (2000 rpm, 10 min). Levels of VEGF-A (446504, Biolegend) in cell supernatants were determined according to the manufacturer’s protocol.

### Tubulogenesis assay

For tubulogenesis assay, wells of a 96-well plate were coated with 40 µL of Cultrex Reduced Growth Factor Basement Membrane Extract (Type R1, # 3433-010-R1, Bio-techne) and allowed to polymerize for 30 min at 37 °C. 15,000 HUVECs were seeded per well with bCAFs CM (generated with the same protocol as for ELISA) for 6 h. For experiments with VEGF inhibitor (Bevacizumab, Avastin), bCAFs CM were pre-incubated with antibody at 2 mg/mL for 2 h at 37 °C before the tubulogenesis assay. The images were acquired on an EVOS XLCore microscope with a 4× objective. For tube quantifications, images were processed in a blind manner using ImageJ software (NIH). All conditions are performed in duplicate and the whole wells area was used for quantification. A representative area of interest of well was illustrated in the figures.

### Chick embryo Chorioallantoic membrane (CAM) model

Fertilized chicken eggs were purchased from EARL LES BRUYERES (28190 DANGERS, France) and incubated for 9 days at 37 °C at 55% relative humidity. At day 9 of their embryonic development (ED9), a window of an ~1 cm-diameter was drilled on top of the air chamber of the eggshell. At ED10, 1 × 10^6^ T47D cell line and 1 × 10^6^ bCAFs (ratio 1:1) were suspended in 25 μL DMEM medium containing 1% fetal bovine serum, 100 U/mL penicillin and streptomycin, and 25 μL Cultrex Reduced Growth Factor Basement Membrane Extract (Type R1, # 3433-010-R1, Bio-techne). The mix was incubated for 15 min at 37 °C and subsequently implanted into the CAM of each egg. The tumors were treated with a VEGF neutralizing antibody (Bevacizumab, 100 µg/CAM) at 3 and 5 days after inoculation. Seven days after implantation (ED17), chick embryos were sacrificed by decapitation. For experiments with chemotherapy, bCAFs were pre-treated during 18 h with chemotherapy in DMEM supplemented with 1% of FBS before their xenograft in CAM. At the end of experiments, tumors were excised, photographed with a Canon EOS 250D camera and carefully weighed to determine their mass. The vascular density within a 5 mm radius around the tumors was quantified on Fiji software using the formula: vessel area/total area × 100%.

### Cytochrome C release

bCAFs were fixed and permeabilized using FIX & PERM Cell Fixation and Permeabilization Kits (00-5523-00, ebioscience, San Jose, CA, USA) for 15 min—RT. After PBS wash and centrifugation, cell suspension was incubated with AlexaFluor®488-conjugated human Cyt-C antibody (BD Pharmingen, 560263, 1/50) for 45 min—RT. Alexa Fluor® 488 Mouse IgG1, κ Isotype Ctrl (Fc) Antibody (Biolegend, 400129) was used at the same concentration. Fluorescence intensity was measured on BD Accuri™ C6 (BD Biosciences) and analyzed with FlowJo software.

### Immunocytochemistry

Cells were fixed in PBS containing 4% paraformaldehyde/4% sucrose for 15 min. Cells were permeabilized for 7 min at RT in 0.25% Triton-X-100 in PBS, washed twice with PBS, and blocked for 45 min at RT in PBS containing 10% BSA. Cells were incubated overnight at 4 °C with NFkB-p65 (Cell Signaling, 8242, 1/200) diluted in PBS containing 3% BSA. After washing, cells were incubated for 45 min at 37 °C with the appropriate Alexa 546-conjugated secondary antibody diluted in PBS containing 3% BSA. Cells were washed with PBS, incubated with DAPI diluted in PBS (Invitrogen, 1/1000) for 10 min and mounted with ProLong Diamond Antifade Reagent (Invitrogen, Carlsbad, CA, USA). Fluorescence images were acquired with Nikon A1 Rsi Inverted Confocal Microscope (Nikon, Tokyo, Japan) with NIS-Elements software (Nikon). Images set position were generated randomly. Around fifty to one hundred fifty cells were analysed per condition. Counting events for nuclear NFkB (NFkB^nuc^ > NFkB^cyto^) were done manually through NIS-Elements software (Nikon).

### Statistical analysis

Student’s *t*-test was used for statistical analysis in experiments with two groups, one sample *t*-test for statistical analysis in experiments with one group and Two-way analysis of variance (ANOVA) was used for statistical analysis for overall condition effects with GraphPad Prism 10.2 Software. Spearman’s correlation test was used for correlations. All data are presented as mean ± SEM of at least three independent experiments. The symbols correspond to a *P*-value inferior to *0.05, **0.01, ***0.001, and ****0.0001.

## Supplementary information

supplementary material revised

Supplementary Figure 1

Supplementary Figure 2

Supplementary Figure 3

Supplementary Figure 4

Supplementary table 1

Original western blot

## Data Availability

The data that support the findings of this study are available from the corresponding author, [FS], upon reasonable request. The mass spectrometry proteomics data are available via ProteomeXchange with identifier PXD058906. The sc-RNAseq data are available, accession number GSE299144.

## References

[1] Yamaguchi K, Hara Y, Kitano I, Hamamoto T, Kiyomatsu K, Yamasaki F, et al. Tumor-stromal ratio (TSR) of invasive breast cancer: correlation with multi-parametric breast MRI findings. Br J Radiol Mai. 2019;92:20181032. 10.1259/bjr.20181032 PMC6580921 30835501

[2] Yan D, Ju X, Luo B, Guan F, He H, Yan H, et al. Tumour stroma ratio is a potential predictor for 5-year disease-free survival in breast cancer. BMC Cancer. 2022;22:1082. 36271354 10.1186/s12885-022-10183-5 PMC9585868

[3] Le MK, Odate T, Kawai M, Oishi N, Kondo T. Investigating the role of core needle biopsy in evaluating tumor-stroma ratio (TSR) of invasive breast cancer: a retrospective study. Breast Cancer Res Treat. 2023;197:113–21. 36335529 10.1007/s10549-022-06768-0

[4] Gaggioli C, Hooper S, Hidalgo-Carcedo C, Grosse R, Marshall JF, Harrington K, et al. Fibroblast-led collective invasion of carcinoma cells with differing roles for RhoGTPases in leading and following cells. Nat Cell Biol. 2007;9:1392–400. 18037882 10.1038/ncb1658

[5] De Wever O, Demetter P, Mareel M, Bracke M. Stromal myofibroblasts are drivers of invasive cancer growth. Int J Cancer. 2008;123:2229–38. 18777559 10.1002/ijc.23925

[6] Costa A, Kieffer Y, Scholer-Dahirel A, Pelon F, Bourachot B, Cardon M, et al. Fibroblast heterogeneity and immunosuppressive environment in human breast cancer. Cancer Cell. 2018;33:463–479.e10. 29455927 10.1016/j.ccell.2018.01.011

[7] Kieffer Y, Hocine HR, Gentric G, Pelon F, Bernard C, Bourachot B, et al. Single-cell analysis reveals fibroblast clusters linked to immunotherapy resistance in cancer. Cancer Discov. 2020;10:1330–51. 32434947 10.1158/2159-8290.CD-19-1384

[8] Croizer H, Mhaidly R, Kieffer Y, Gentric G, Djerroudi L, Leclere R, et al. Deciphering the spatial landscape and plasticity of immunosuppressive fibroblasts in breast cancer. Nat Commun. 2024;15:2806. 38561380 10.1038/s41467-024-47068-z PMC10984943

[9] De Francesco EM, Lappano R, Santolla MF, Marsico S, Caruso A, Maggiolini M. HIF-1α/GPER signaling mediates the expression of VEGF induced by hypoxia in breast cancer associated fibroblasts (CAFs). Breast Cancer Res. 2013;15:R64. 23947803 10.1186/bcr3458 PMC3978922

[10] Inoue K, Kishimoto S, Akimoto K, Sakuma M, Toyoda S, Inoue T, et al. Cancer-associated fibroblasts show heterogeneous gene expression and induce vascular endothelial growth factor A (VEGFA) in response to environmental stimuli. Ann Gastroenterol Surg. 2019;3:416–25. 31346581 10.1002/ags3.12249 PMC6635680

[11] Wang FT, Sun W, Zhang JT, Fan YZ. Cancer-associated fibroblast regulation of tumor neo-angiogenesis as a therapeutic target in cancer. Oncol Lett. 2019;17:3055–65. 30867734 10.3892/ol.2019.9973 PMC6396119

[12] Busch S, Acar A, Magnusson Y, Gregersson P, Rydén L, Landberg G. TGF-beta receptor type-2 expression in cancer-associated fibroblasts regulates breast cancer cell growth and survival and is a prognostic marker in pre-menopausal breast cancer. Oncogene. 2015;34:27–38. 24336330 10.1038/onc.2013.527

[13] Kadel D, Zhang Y, Sun HR, Zhao Y, Dong QZ, Qin Lxiu. Current perspectives of cancer-associated fibroblast in therapeutic resistance: potential mechanism and future strategy. Cell Biol Toxicol. 2019;35:407–21. 30680600 10.1007/s10565-019-09461-z PMC6881418

[14] Erdogan B, Webb DJ. Cancer-associated fibroblasts modulate growth factor signaling and extracellular matrix remodeling to regulate tumor metastasis. Biochem Soc Trans. 2017;45:229–36. 28202677 10.1042/BST20160387 PMC5371349

[15] Pelon F, Bourachot B, Kieffer Y, Magagna I, Mermet-Meillon F, Bonnet I, et al. Cancer-associated fibroblast heterogeneity in axillary lymph nodes drives metastases in breast cancer through complementary mechanisms. Nat Commun. 2020;11:404. 31964880 10.1038/s41467-019-14134-w PMC6972713

[16] Ye F, Liang Y, Wang Y, Le Yang R, Luo D, Li Y, et al. Cancer-associated fibroblasts facilitate breast cancer progression through exosomal circTBPL1-mediated intercellular communication. Cell Death Dis. 2023;14:471. 37495592 10.1038/s41419-023-05986-8 PMC10372047

[17] Juin P, Geneste O, Gautier F, Depil S, Campone M. Decoding and unlocking the BCL-2 dependency of cancer cells. Nat Rev Cancer. 2013;13:455–65. 23783119 10.1038/nrc3538

[18] Louault K, Bonneaud TL, Séveno C, Gomez-Bougie P, Nguyen F, Gautier F, et al. Interactions between cancer-associated fibroblasts and tumor cells promote MCL-1 dependency in estrogen receptor-positive breast cancers. Oncogene. 2019;38:3261–73. 30631150 10.1038/s41388-018-0635-z PMC6756023

[19] Bonneaud TL, Lefebvre CC, Nocquet L, Basseville A, Roul J, Weber H, et al. Targeting of MCL-1 in breast cancer-associated fibroblasts reverses their myofibroblastic phenotype and pro-invasive properties. Cell Death Dis. 2022;13:787. 36104324 10.1038/s41419-022-05214-9 PMC9474880

[20] Chen W, Zhao H, Li Y. Mitochondrial dynamics in health and disease: mechanisms and potential targets. Sig Transduct Target Ther. 2023;8:333. 10.1038/s41392-023-01547-9 PMC10480456 37669960

[21] Giampazolias E, Zunino B, Dhayade S, Bock F, Cloix C, Cao K, et al. Mitochondrial permeabilization engages NF-κB-dependent anti-tumour activity under caspase deficiency. Nat Cell Biol. 2017;19:1116–29. 28846096 10.1038/ncb3596 PMC5624512

[22] Vringer E, Heilig R, Riley JS, Black A, Cloix C, Skalka G, et al. Mitochondrial outer membrane integrity regulates a ubiquitin-dependent and NF-κB-mediated inflammatory response. EMBO J. 2024;43:904–30. 38337057 10.1038/s44318-024-00044-1 PMC10943237

[23] Harding O, Holzer E, Riley JF, Martens S, Holzbaur ELF. Damaged mitochondria recruit the effector NEMO to activate NF-κB signaling. Molecular Cell. 2023;83:3188–3204.e7. 37683611 10.1016/j.molcel.2023.08.005 PMC10510730

[24] Kotschy A, Szlavik Z, Murray J, Davidson J, Maragno AL, Le Toumelin-Braizat G, et al. The MCL1 inhibitor S63845 is tolerable and effective in diverse cancer models. Nature. 2016;538:477–82. 27760111 10.1038/nature19830

[25] Amini A, Masoumi Moghaddam S, Morris DL, Pourgholami MH. The critical role of vascular endothelial growth factor in tumor angiogenesis. Curr Cancer Drug Targets. 2012;12:23–43. 22111836 10.2174/156800912798888956

[26] Melincovici CS, Boşca AB, Şuşman S, Mărginean M, Mihu C, Istrate M, et al. Vascular endothelial growth factor (VEGF) - key factor in normal and pathological angiogenesis. Rom J Morphol Embryol. 2018;59:455–67. 30173249

[27] Ghalehbandi S, Yuzugulen J, Pranjol MZI, Pourgholami MH. The role of VEGF in cancer-induced angiogenesis and research progress of drugs targeting VEGF. European J Pharmacol. 2023;949:175586. 36906141 10.1016/j.ejphar.2023.175586

[28] Miebach L, Berner J, Bekeschus S. In ovo model in cancer research and tumor immunology. Front Immunol. 2022;13:1006064. 36248802 10.3389/fimmu.2022.1006064 PMC9556724

[29] Kim J, Kim HS, Chung JH. Molecular mechanisms of mitochondrial DNA release and activation of the cGAS-STING pathway. Exp Mol Med. 2023;55:510–9. 36964253 10.1038/s12276-023-00965-7 PMC10037406

[30] Albert MC, Brinkmann K, Kashkar H. Noxa and cancer therapy: tuning up the mitochondrial death machinery in response to chemotherapy. Mol Cell Oncol. 2014;1:e29906. 27308315 10.4161/mco.29906 PMC4905168

[31] Czabotar PE, Lee EF, van Delft MF, Day CL, Smith BJ, Huang DCS, et al. Structural insights into the degradation of Mcl-1 induced by BH3 domains. Proc Natl Acad Sci USA. 2007;104:6217–22. 17389404 10.1073/pnas.0701297104 PMC1851040

[32] Haschka MD, Soratroi C, Kirschnek S, Häcker G, Hilbe R, Geley S, et al. The NOXA–MCL1–BIM axis defines lifespan on extended mitotic arrest. Nat Commun. 2015;6:6891. 25922916 10.1038/ncomms7891 PMC4423218

[33] Arai S, Varkaris A, Nouri M, Chen S, Xie L, Balk SP. MARCH5 mediates NOXA-dependent MCL1 degradation driven by kinase inhibitors and integrated stress response activation. Elife. 2020;9:e54954. 32484436 10.7554/eLife.54954 PMC7297531

[34] Beroukhim R, Mermel CH, Porter D, Wei G, Raychaudhuri S, Donovan J, et al. The landscape of somatic copy-number alteration across human cancers. Nature. 2010;463:899–905. 20164920 10.1038/nature08822 PMC2826709

[35] Rasmussen ML, Kline LA, Park KP, Ortolano NA, Romero-Morales AI, Anthony CC, et al. A non-apoptotic function of MCL-1 in promoting pluripotency and modulating mitochondrial dynamics in stem cells. Stem Cell Rep. 2018;10:684–92. 10.1016/j.stemcr.2018.01.005 PMC5918190 29429957

[36] Song H, Lu T, Han D, Zhang J, Gan L, Xu C, et al. YAP1 inhibition induces phenotype switching of cancer-associated fibroblasts to tumor suppressive in prostate cancer. Cancer Res. 2024;84:3728–42. 39137404 10.1158/0008-5472.CAN-24-0932 PMC11565174

[37] Bolesta E, Pfannenstiel LW, Demelash A, Lesniewski ML, Tobin M, Schlanger SE, et al. Inhibition of Mcl-1 promotes senescence in cancer cells: implications for preventing tumor growth and chemotherapy resistance. Mol Cell Biol. 2012;32:1879–92. 22451485 10.1128/MCB.06214-11 PMC3347419

[38] Demelash A, Pfannenstiel LW, Tannenbaum CS, Li X, Kalady MF, DeVecchio J, et al. Structure-function analysis of the Mcl-1 protein identifies a novel senescence-regulating domain. J. Biol Chem. 2015;290:21962–75. 26205817 10.1074/jbc.M115.663898 PMC4571950

[39] Chembukavu SN, Lindsay AJ. Therapy-induced senescence in breast cancer: an overview. Explor Target Anti-tumor Ther. 2024;5:902–20. 10.37349/etat.2024.00254 PMC11390292 39280248

[40] Coppé JP, Patil CK, Rodier F, Sun Y, Muñoz DP, Goldstein J, et al. Senescence-associated secretory phenotypes reveal cell-nonautonomous functions of oncogenic RAS and the p53 tumor suppressor. Downward J, éditeur. PLoS Biol. 2008;6:e301. 19053174 10.1371/journal.pbio.0060301 PMC2592359

[41] Weidner N, Semple JP, Welch WR, Folkman J. Tumor angiogenesis and metastasis–correlation in invasive breast carcinoma. N Engl J Med. 1991;324:1–8. 1701519 10.1056/NEJM199101033240101

[42] Weidner N, Folkman J, Pozza F, Bevilacqua P, Allred EN, Moore DH, et al. Tumor angiogenesis: a new significant and independent prognostic indicator in early-stage breast carcinoma. J Natl Cancer Inst. 1992;84:1875–87. 1281237 10.1093/jnci/84.24.1875

[43] Abbasi A, Ghaffarizadeh F, Mojdeganlou H. Prognostic significance of microvessel density in invasive ductal carcinoma of breast. Int J Hematol Oncol Stem Cell Res. 2023;17:100–5. 37637763 10.18502/ijhoscr.v17i2.12646 PMC10452950

[44] De Bock K, Georgiadou M, Schoors S, Kuchnio A, Wong BW, Cantelmo AR, et al. Role of PFKFB3-driven glycolysis in vessel sprouting. Cell. 2013;154:651–63. 23911327 10.1016/j.cell.2013.06.037

[45] Rappsilber J, Ishihama Y, Mann M. Stop and go extraction tips for matrix-assisted laser desorption/ionization, nanoelectrospray, and LC/MS sample pretreatment in proteomics. Anal Chem. 2003;75:663–70. 12585499 10.1021/ac026117i

[46] Pfaffl MW. A new mathematical model for relative quantification in real-time RT-PCR. Nucleic Acids Res. 2001;29:45e–45. 10.1093/nar/29.9.e45 PMC55695 11328886

