# Supplementary material for: MCL-1 as a molecular switch between myofibroblastic and pro-angiogenic features of breast cancer-associated fibroblasts
Source: Cell Death Dis. 2025 Aug 9;16(1):603. doi: 10.1038/s41419-025-07920-6 (PMC12335522; doi:10.1038/s41419-025-07920-6)
Supplement: Supplementary file 1 — supplementary material revised [file 41419_2025_7920_MOESM1_ESM.docx]

**Supplementary METHODS AND MATERIALS**

**Apoptosis assay**

bCAFs were seeded in a 12-well plate (40 000 cells/well) and were treated as indicated during 18h in DMEM 1% FBS. Cells were washed with PBS after 18h-treatment and cultured in EGM2 supplemented with 1% FBS for additional 72 hours. Then, cell death of bCAFs was assessed using an Annexin-V FITC binding assay (Miltenyi #130-092-052) performed according to manufacturer’s instructions. Flow-cytometry analysis was performed on Accuri C6 Plus flow cytometer (BD Biosciences).

**Proliferation assay**

bCAFs were seeded in a 12-well plate (15 000 cells/well) and were treated as indicated during 18h in DMEM 1% FBS. After rinsing with PBS, cells were incubated for 20min at 37 °C with a solution of 2.5µM CFSE CellTrace™ diluted in PBS (C34554, Thermo Fisher). Then, cells were rinsed with PBS and incubated with DMEM supplemented with 10% FBS for 20 min at 37 °C to absorb excess dye. Cells were finally incubated with DMEM supplemented with 10% FBS and CFSE CellTrace™ intensity was measured on Accuri C6 Plus flow cytometer (BD Biosciences) every other day until day 8 after treatment and analyzed with FlowJo software.

**Cleaved caspase 3 immunostaining**

bCAFs were fixed and permeabilized using FIX & PERM Cell Fixation and Permeabilization Kits (00-5523-00, ebioscience, San Jose, CA, USA) for 15min – RT. After PBS wash and centrifugation, cell suspension was incubated with cleaved caspase 3 primary antibody (Cell Signaling, 9661, 1/100) for 30min – RT. After PBS wash and centrifugation, cell suspension was incubated with the appropriate Alexa 488-conjugated secondary antibody (A11008, Thermo, 1/300) for 30min – RT. Fluorescence intensity was measured on BD Accuri™ C6 (BD Biosciences) and analyzed with FlowJo software.

**Supplementary legends**

**Supplementary figure 1: A.** Enrichment analysis of gene sets preferentially expressed in clusters 0-3 in CAFs sgCTRL. Normalized enrichment scores and adjusted p-values were extracted per cluster, results were visualized as a heatmap displaying NES values, with significance annotations (* p ≤ 0.05, ** p ≤ 0.015, *** p ≤ 0.001). **B.** Dot plots of upregulated secreted factors (log2FC > 0.3, q-value < 0.01) in each cluster after MCL-1 gene silencing in bCAFs. The size of each dot represents the percentage of cells expressing the gene within the cluster, while the color intensity indicates the average expression level.

**Supplementary figure 2: Targeting of Bcl-XL doesn't modulate the pro-angiogenic factors secretion by bCAFs. A.** Bcl-XL protein expression level in bCAFs after gene silencing evaluated using western blots. Actin expression was used as loading control. **B.** qRT-PCR of VEGF-A, FGF2 and ANGPT1 mRNA in bCAF expressing BCL-Xl (sgCTRL) or not (sgBCL-Xl) normalized on GAPDH mRNA expression (n=3). Student t-test, *P < 0.05, ns: not significant. **C.** VEGF-A quantification by ELISA in conditioned media (CM) from bCAFs after Bcl-XL gene silencing (bCAFsgBcl-XL) or not (bCAFsgCTRL). The bCAFs CM were generated during 72h in EGM2 (Endothelial Cell Growth Medium-2) medium supplemented with 1% of FBS (n=3). Student t-test, ns: non-significant. **D.** qRT-PCR of VEGF-A, FGF2 and ANGPT1 mRNA in bCAFs treated or not by A1331852 100 nM for 18 h normalized on GAPDH mRNA expression (n=3). Student t-test, ns: not significant. **E.** VEGF-A quantification by ELISA in CM of bCAFs treated or not by A1331852 100 nM for 18 h Results were expressed as concentration (pg/ml) for 200 000 cells (n=4). Student t-test, ns: non-significant. **F.** Correlation (Spearman correlation coefficient r = −0.04762; p value = 0.9349) between VEGF-A concentration level in bCAFs CM and BCL-Xl protein level (relative to actin level) determined by western-blot analysis in 8 primary cultures of bCAFs between passage 2 and 4.

**Supplementary figure 3:** **A.** STING protein expression level in bCAFs after gene silencing evaluated using western blots. Actin expression was used as loading control (n=3). **B.** qRT-PCR of VEGF-A, CXCL8, IL-1β and CXCL1 mRNA in bCAFs expressing STING (CAFsgCTRL) or not (CAFsgSTING) treated or not with S63845 for 18h normalized on RPLPO mRNA expression (n=3). Two-way ANOVA, ****P < 0.0001, ***P < 0.001, **P < 0.01, ns: not significant. **C.** TBK1 protein expression level in bCAFs after 3h treatment with ProtacCTRL or ProtacTBK1 evaluated using western blots. Actin expression was used as loading control (n=4). **D.** qRT-PCR of VEGF-A, CXCL8, IL-1β and CXCL1 mRNA in bCAFs after treatment for 3h with ProtacCTRL or ProtacTBK1 before adding treatment for 18h with S63845 500nM normalized on RPLPO mRNA expression (n=4). Two-way ANOVA, ****P < 0.0001, ***P < 0.001, **P < 0.01, ns: not significant. **E.** TBK1 and MCL-1 protein expression levels in bCAFs after ProtacTBK1 or ProtacCTRL treatment for 3h before adding S63845 for 18h evaluated using western blots. Actin expression was used as loading control.

**Supplementary figure 4:** **A.** Apoptotic cell death of bCAFs after chemotherapy (1µM) for 18h in DMEM containing 1% FBS was measured by Annexin-V flow cytometry assay (n=6), Student t-test, **P<0.01. **B.** Apoptotic cell death of bCAFs treated with chemotherapy (1µM) or S63845 (500nM) for 18h in DMEM containing 1% FBS followed by 72h-conditioning in EGM2 supplemented with 1% FBS was measured by Annexin-V flow cytometry assay (n=4), Student t-test, ns: non-significant. **C.** Cell quantification of bCAFs treated for chemotherapy (1µM) or S63845 (500nM) for 18h in DMEM containing 1% FBS followed by 72h-conditioning in EGM2 supplemented with 1% FBS (n=4), Student t-test, ns: non-significant. **D.** MCL-1 and BCL-xL protein expression levels in bCAFs surexpressing MCL-1 (CAFpLvxMCL1), BCL-xL (CAFpLvxBCL-xL) or not (CAFpLvxCTRL) were evaluated using western blots analysis. Actin expression was used as loading control (n=3). **E.** NOXA protein expression level in bCAFs after gene silencing evaluated using western blots. Actin expression was used as loading control (n=4). **F.** MFI of cleaved caspase 3 in bCAFs after 18h of treatment with S63845 500nM or chemotherapy 1µM in combination or not with pan-caspase inhibitor (Q-VD-OPH 5µM) was measured by flow cytometry. Co-treatment with S63845 (500nM) and A1331852 (BCL-xL inh., 100nM) served as positive control of cleaved caspase 3 (n=3). Two-way ANOVA, ****P < 0.0001, ns: non-significant. **G.** VEGF-A secretion analysed by ELISA in bCAFs CM after chemotherapy (1 µM) in combination with pan-caspase inhibitor (Q-VD-OPH 5µM) (n=4). Two-way ANOVA, *P < 0.05; **P < 0.01; ns: non-significant.
