# Supplementary figures and images for: MCL-1 as a molecular switch between myofibroblastic and pro-angiogenic features of breast cancer-associated fibroblasts
Source: Cell Death Dis. 2025 Aug 9;16(1):603. doi: 10.1038/s41419-025-07920-6 (PMC12335522; doi:10.1038/s41419-025-07920-6)

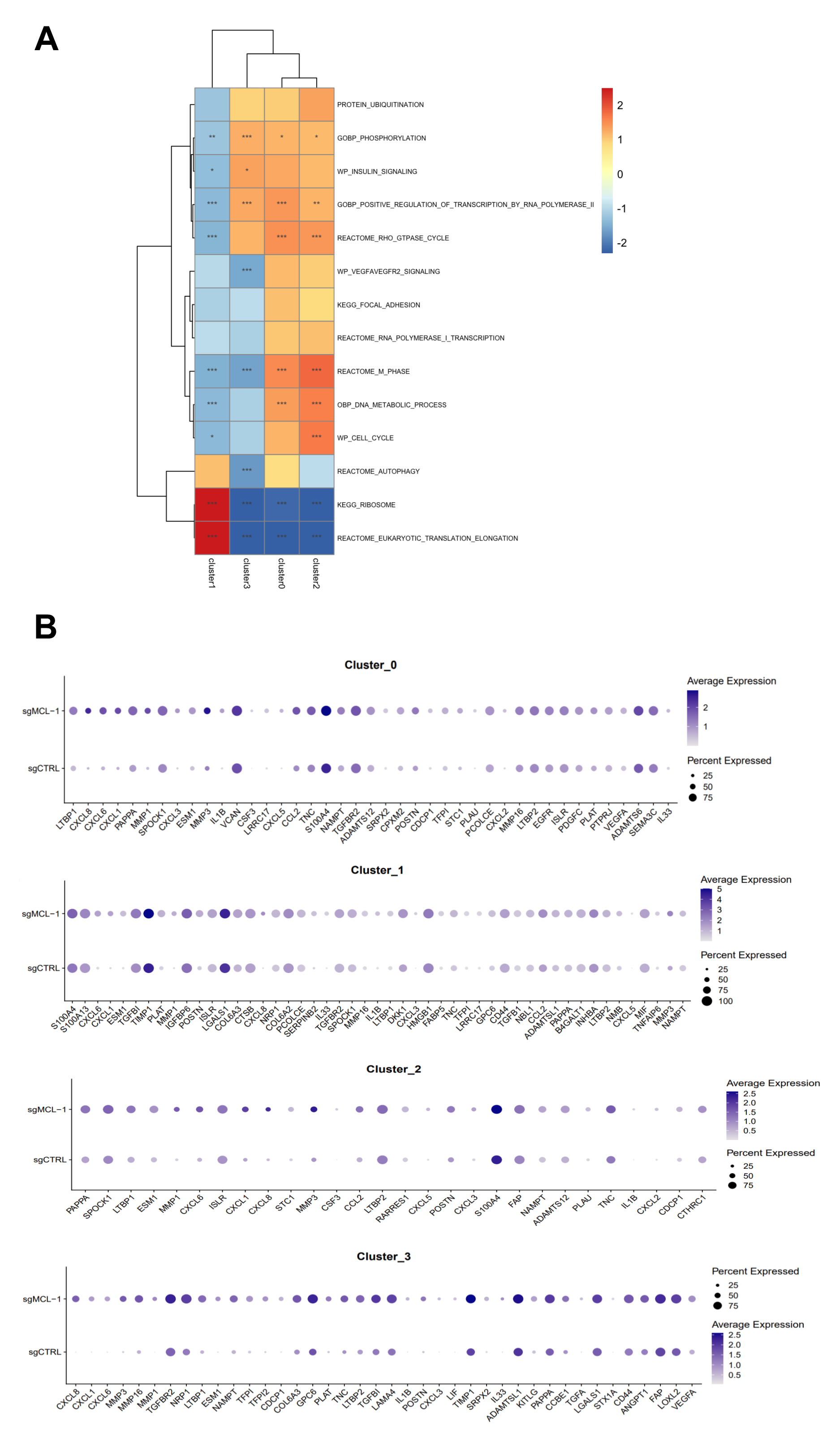

Supplement: Supplementary file 2 — Supplementary Figure 1 [file 41419_2025_7920_MOESM2_ESM.tif]

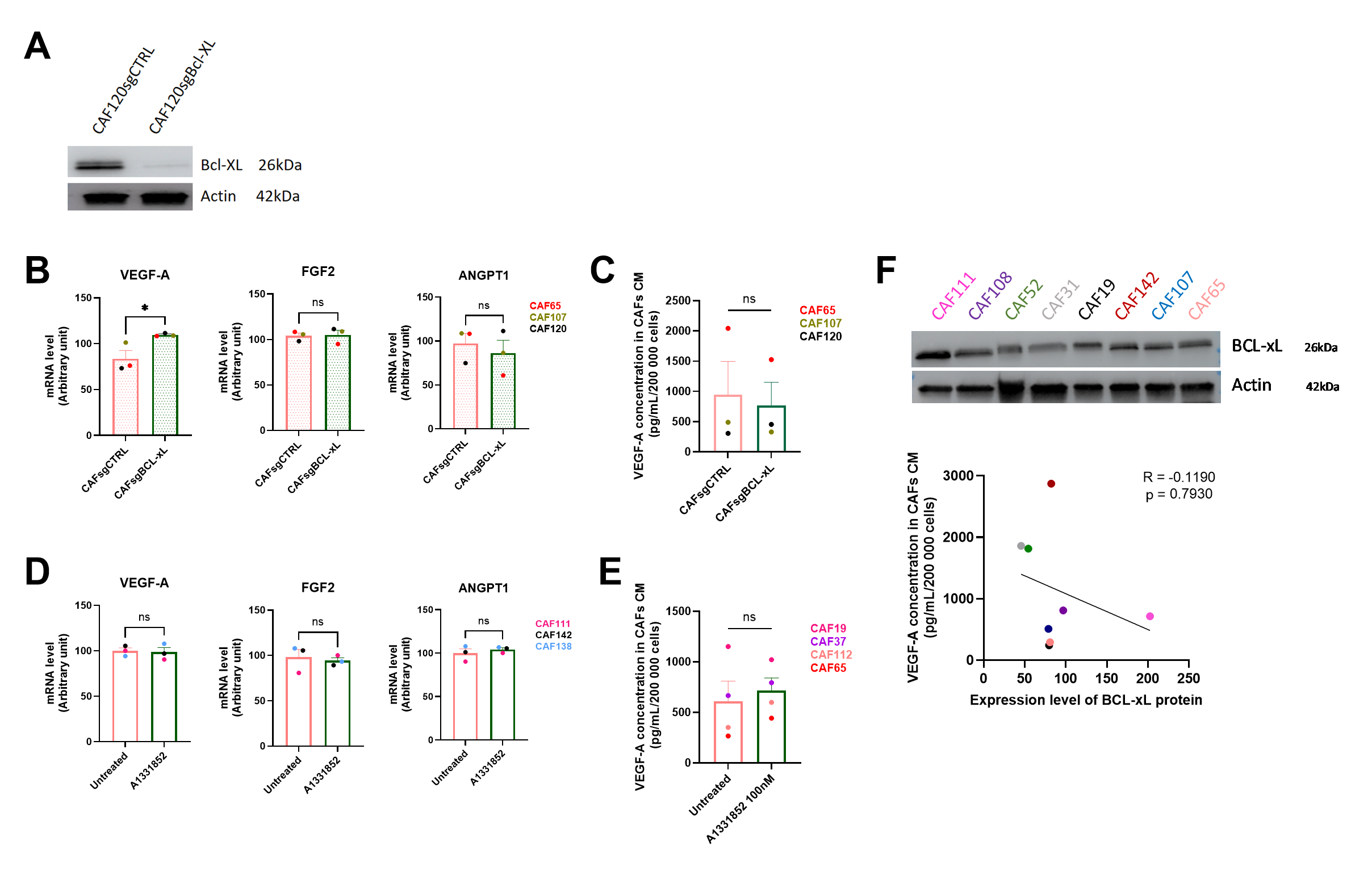

Supplement: Supplementary file 3 — Supplementary Figure 2 [file 41419_2025_7920_MOESM3_ESM.tif]

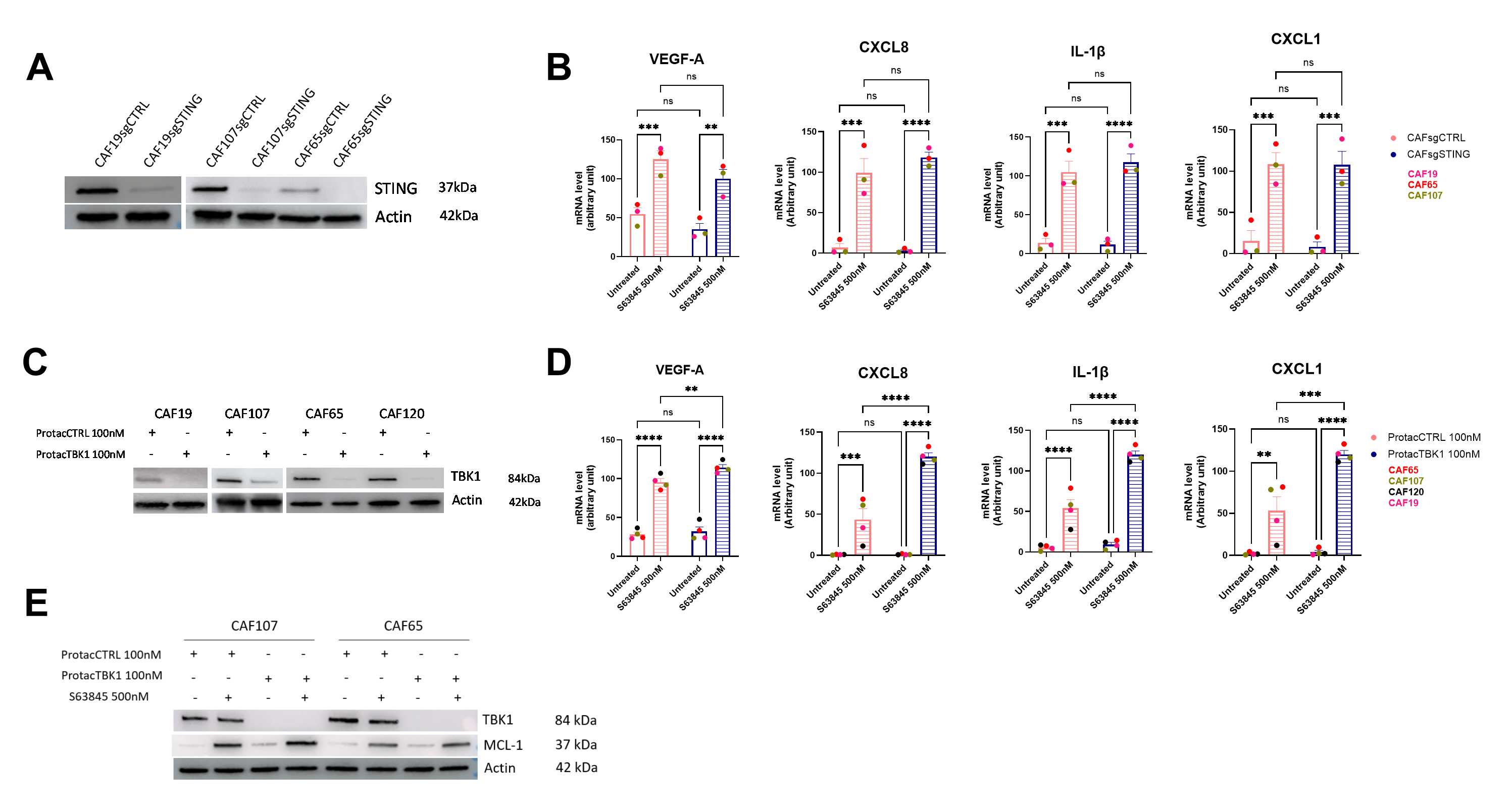

Supplement: Supplementary file 4 — Supplementary Figure 3 [file 41419_2025_7920_MOESM4_ESM.tif]

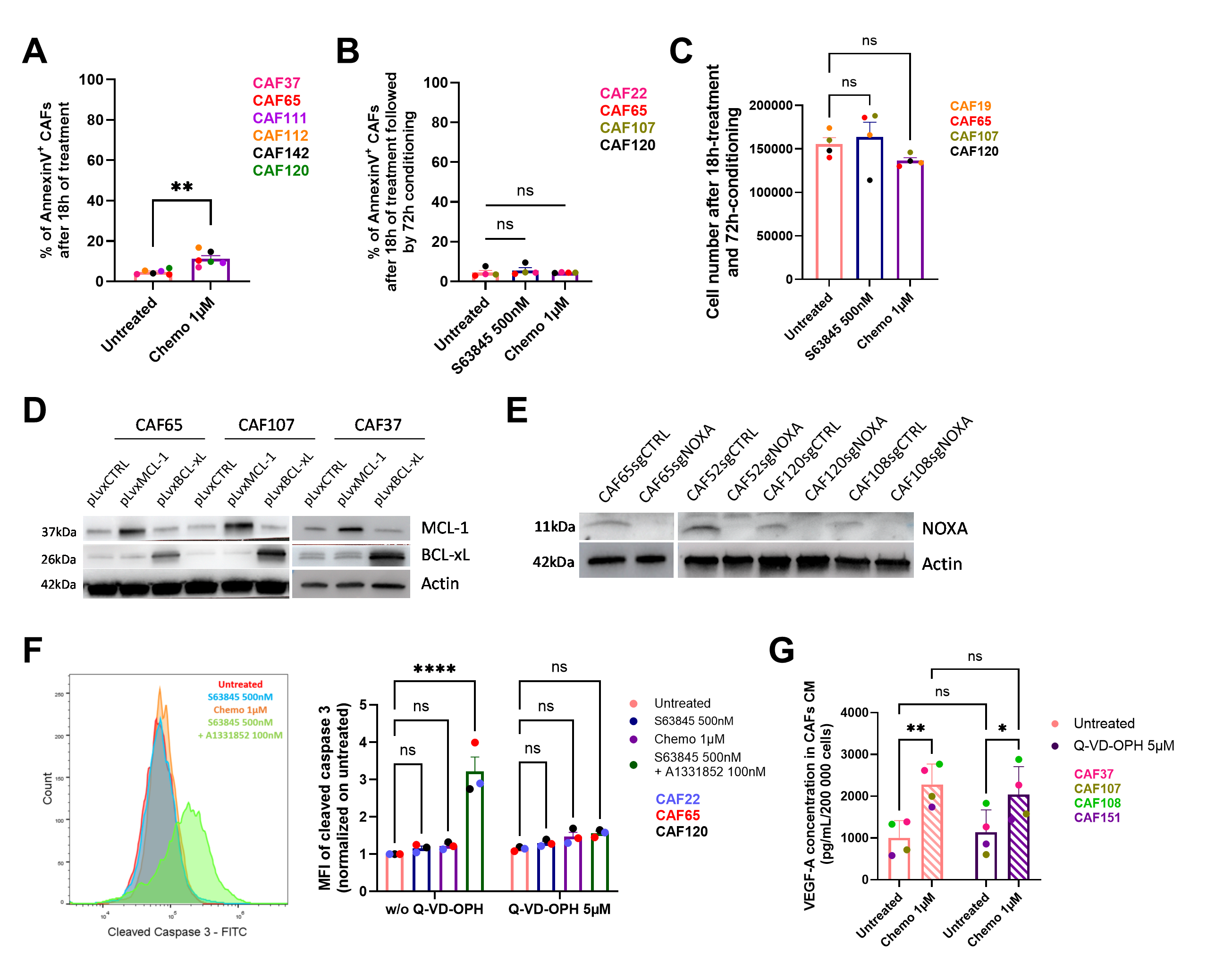

Supplement: Supplementary file 5 — Supplementary Figure 4 [file 41419_2025_7920_MOESM5_ESM.tif]

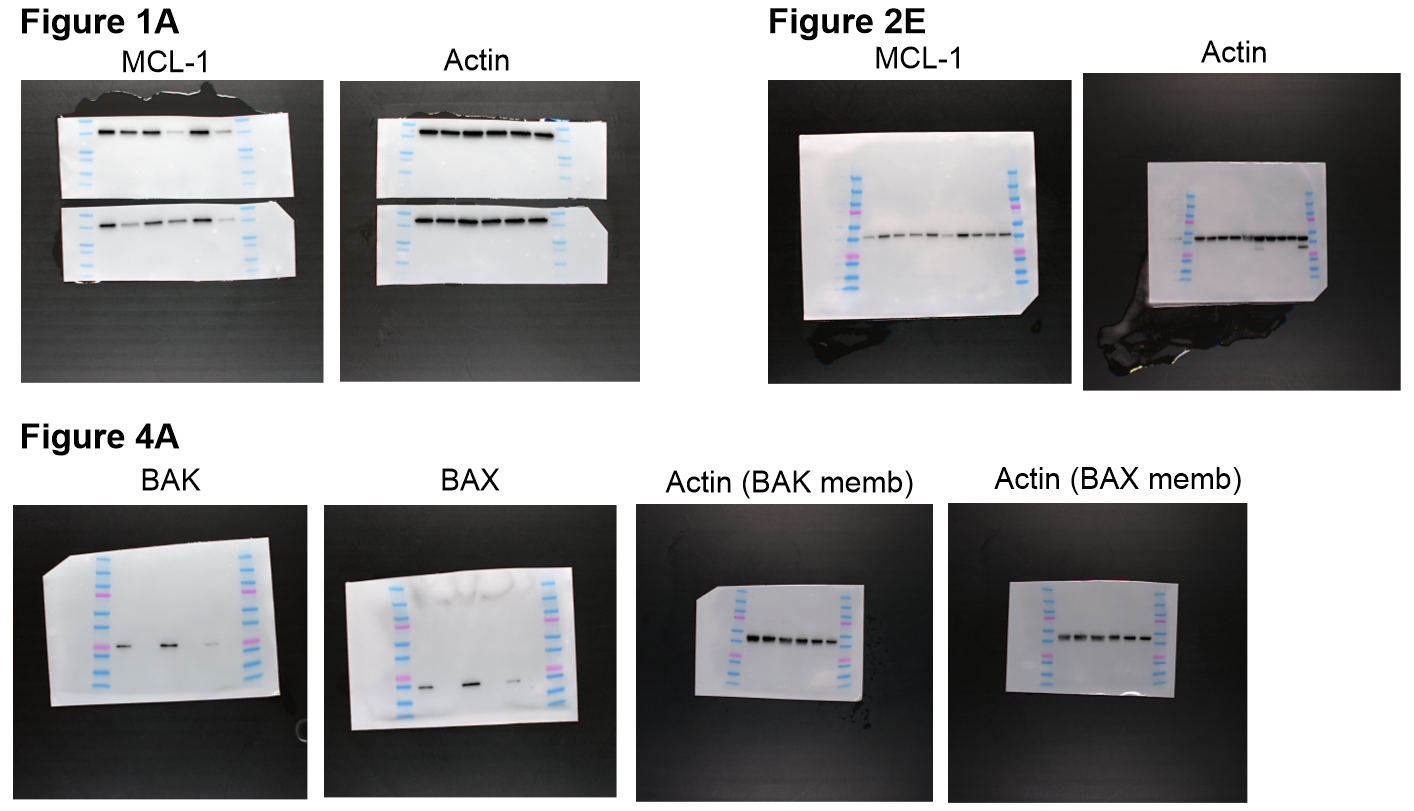


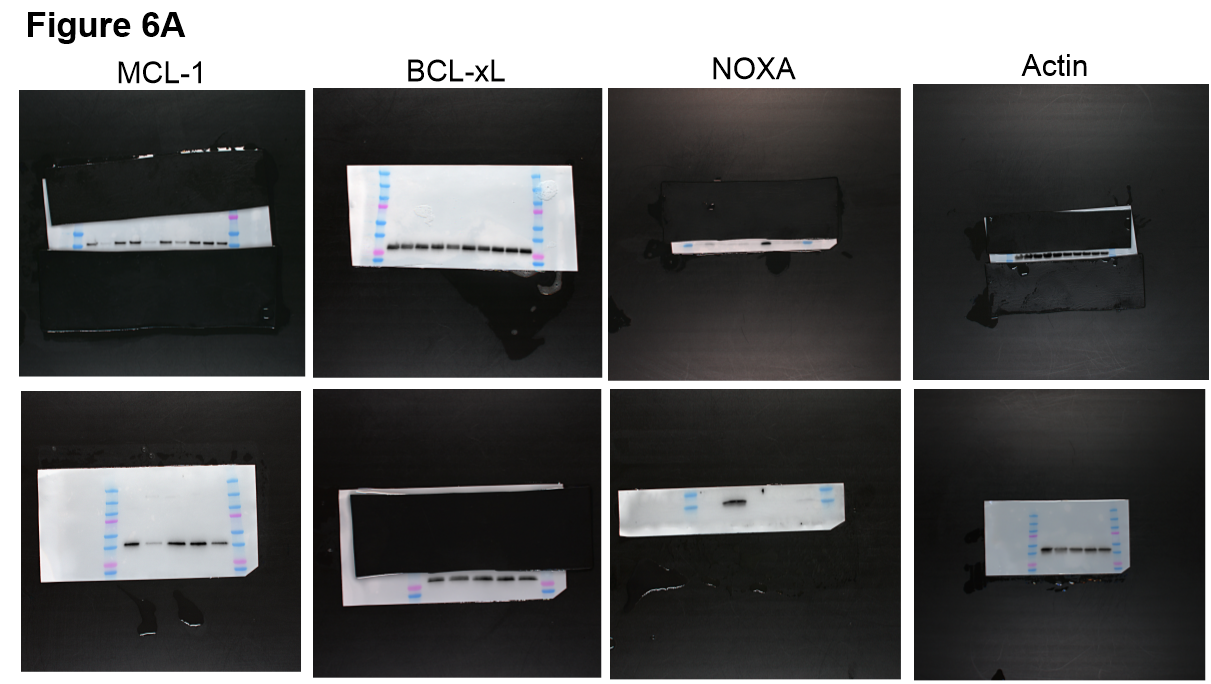


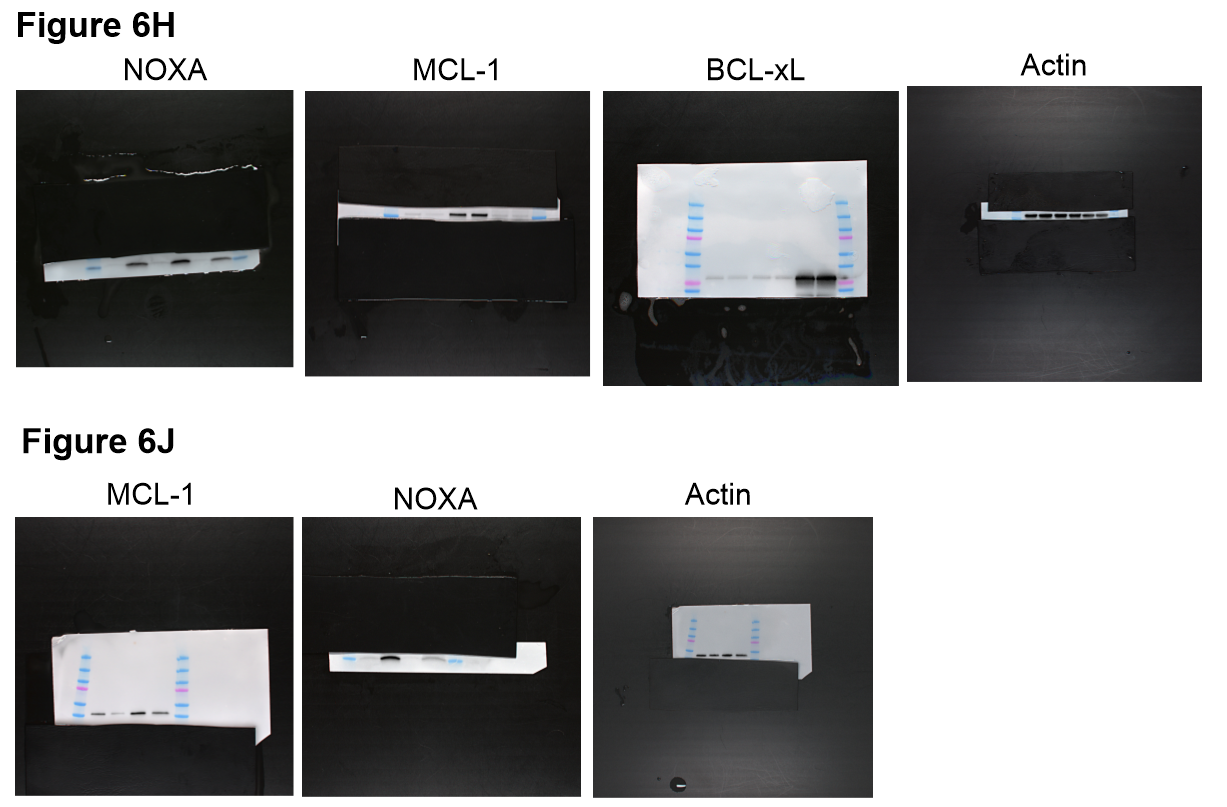


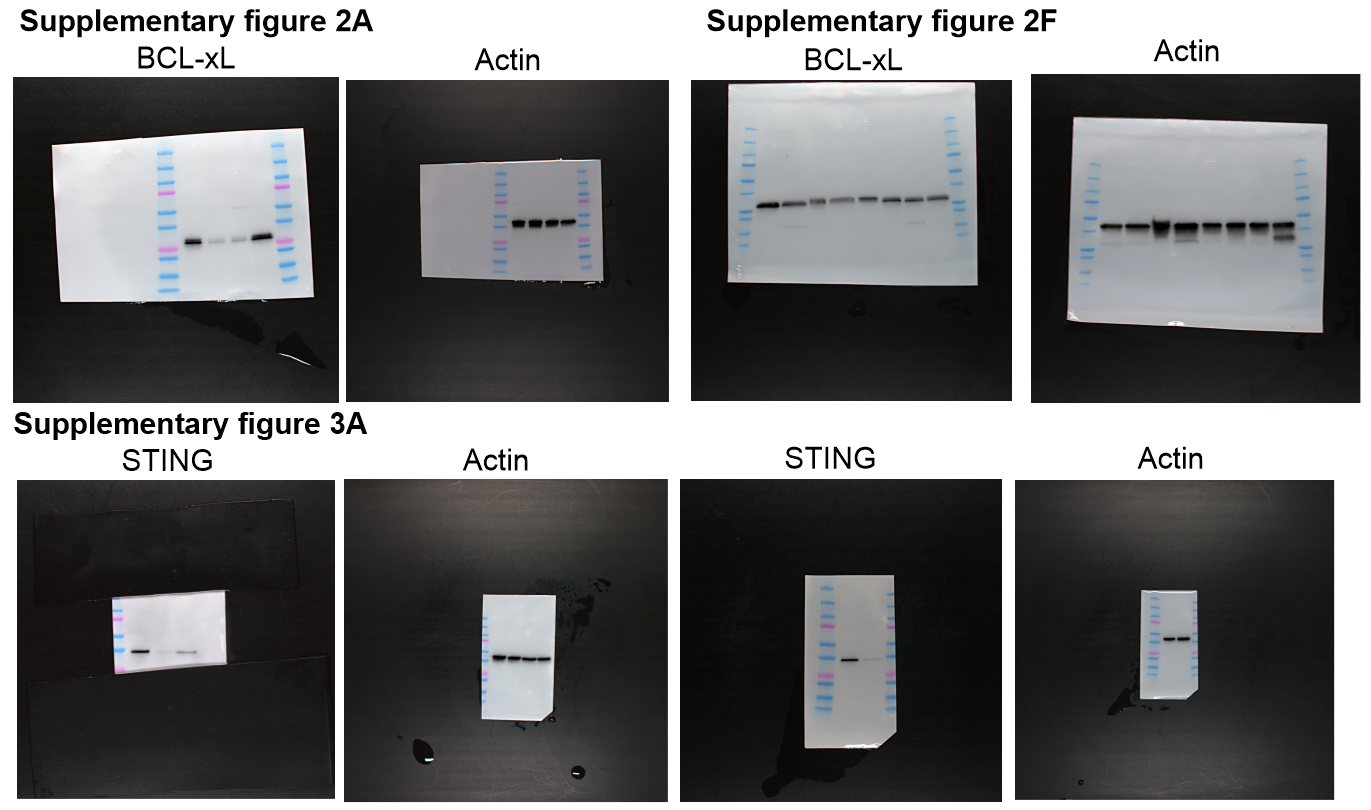


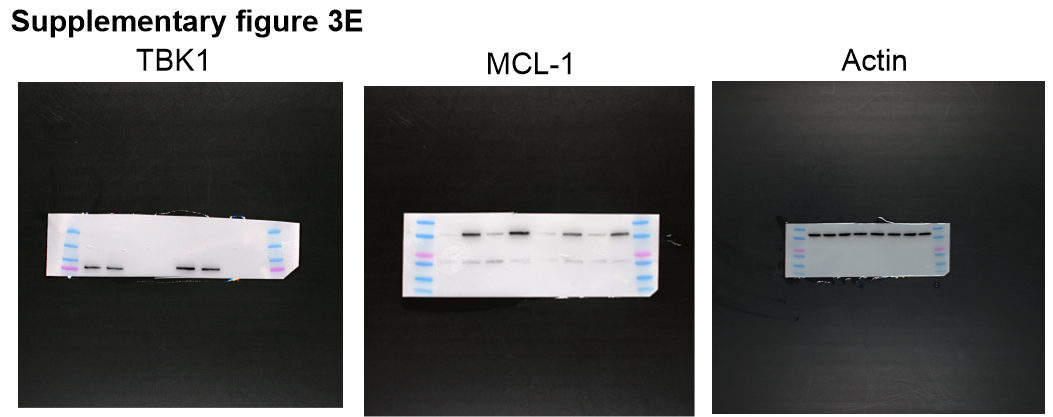

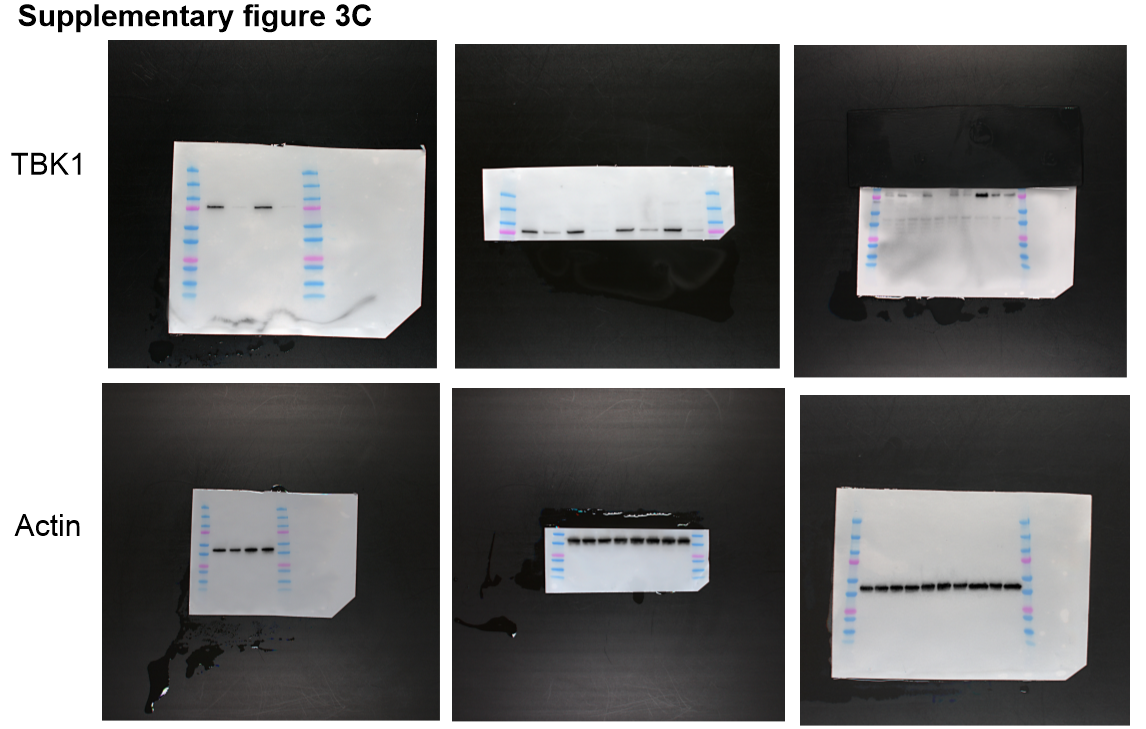


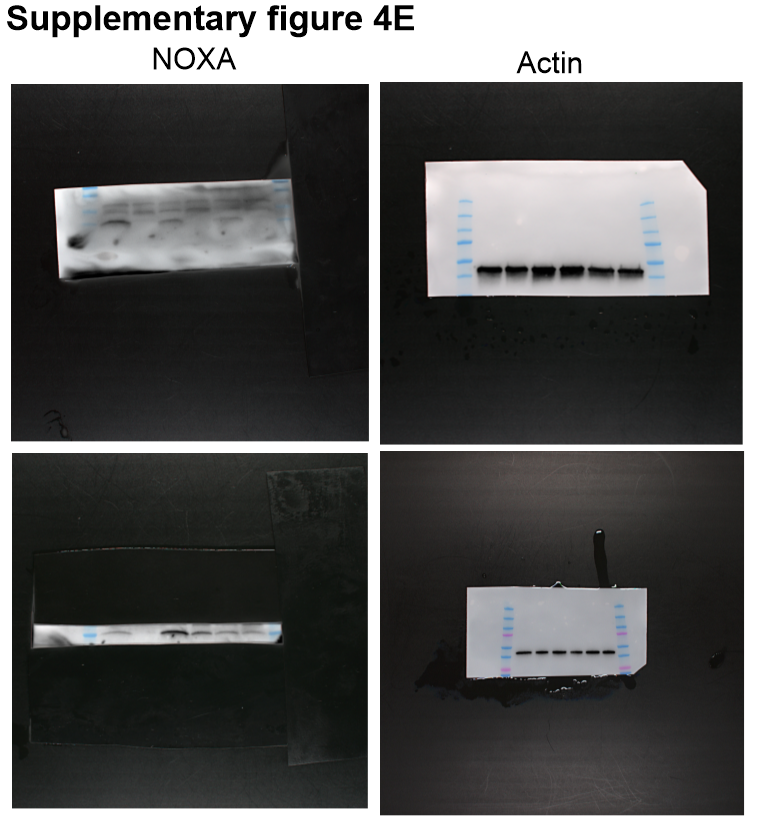

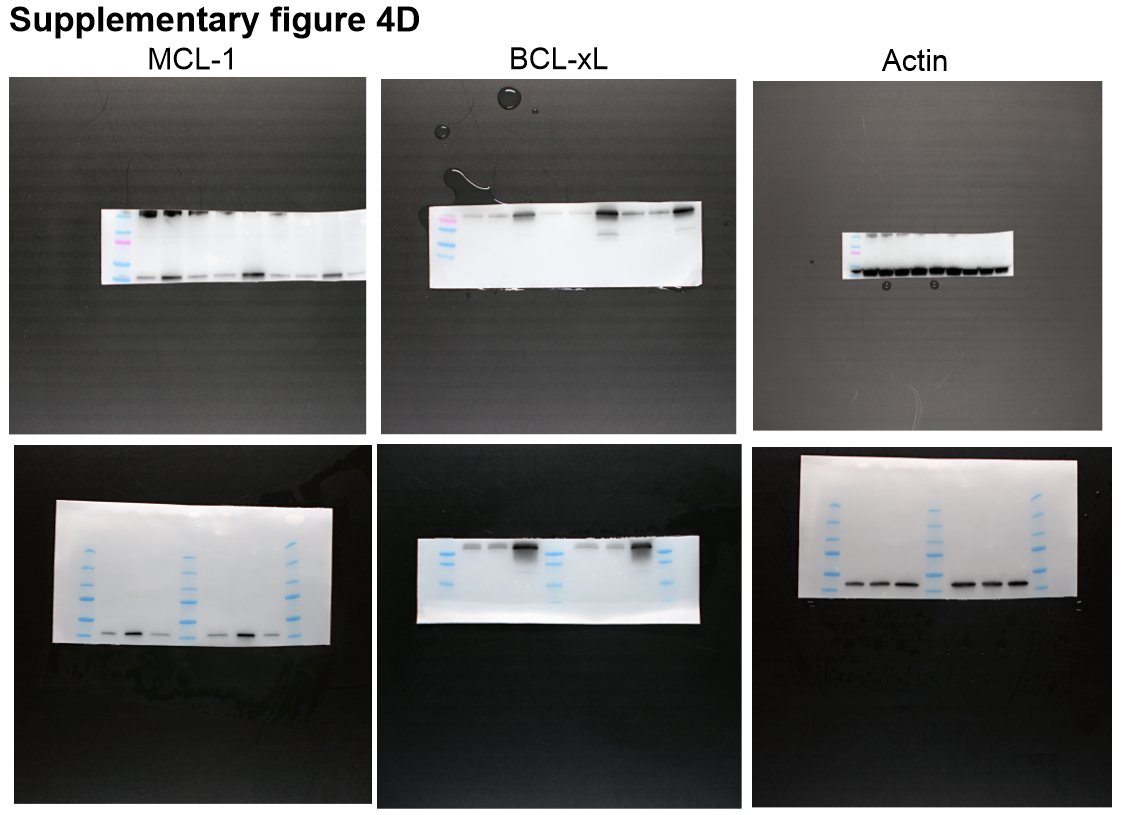

Supplement: Supplementary file 7 — Original western blot [file 41419_2025_7920_MOESM7_ESM.docx]
